# Supplementary material for: Deciphering climate-induced displacement in Somalia: A remote sensing perspective
Source: PLoS One. 2024 Aug 9;19(8):e0304202. doi: 10.1371/journal.pone.0304202 (PMC11315326; doi:10.1371/journal.pone.0304202)

PLOS ONE

Deciphering Climate-induced Displacement in Somalia: A Remote Sensing Perspective

--Manuscript Draft--

**Manuscript Number:**	PONE-D-23-15881
**Article Type:**	Research Article
**Full Title:**	Deciphering Climate-induced Displacement in Somalia:<br>A Remote Sensing Perspective
**Short Title:**	Deciphering Climate-induced Displacement in Somalia:<br>A Remote Sensing Perspective
**Corresponding Author:**	Tuba Bircan<br>Vrije Universiteit Brussel<br>Brussels, BELGIUM
**Keywords:**	Remote Sensing; Satellite Data; Human Migration; Internal Displacement
**Abstract:**	Rapid changes in climate have reflections on all different aspects of our lives, one prominent effect is detreating the living conditions vastly in particular areas where people have no choice but flee. Despite the acknowledgement of this topic, the dynamic of the relationship between the environmental factors and the human mobility is yet to be studied in detail. This study aims to investigate the use of advanced remote sensing analytics for developing advanced climate indicators developed on a micro (district) level using and to study the relationship between climate factors and internally displaced persons. After elaborating on our data sources and the analytics used for indicator development, we discuss different types of events and their aftermath. Our findings confirm that slow-onset climate events and rapid-onset climate events have a different impact on society and the responses depend on the urge due to the deterring aftermath of the extreme weather event and most importantly the capabilities of people. We also emphasise that data quality and availability for the socio-economic indicators are of importance to enhance future studies given the interlinked associations between climate change, economic deprivation, and violent conflict.
**Order of Authors:**	Rahman Momeni
	Tuba Bircan
	Richard King
**Additional Information:**	
**Question**	**Response**
**Financial Disclosure**<br><br>Enter a financial disclosure statement that describes the sources of funding for the work included in this submission. Review the submission guidelines for detailed requirements. View published research articles from *PLOS ONE* for specific examples.<br><br>This statement is required for submission and **will appear in the published article** if the submission is accepted. Please make sure it is accurate.	MR, BT, KR<br><br>This research is supported by the European Commission through the Horizon2020 European project: “HumMingBird – Enhanced migration measures from a multidimensional perspective” (GA: 870661).<br><br>https://research-and-innovation.ec.europa.eu/funding/funding-opportunities/funding-programmes-and-open-calls/horizon-europe_en<br><br>The funders had no role in study design, data collection and analysis, decision to publish, or preparation of the manuscript.

Powered by Editorial Manager® and ProduXion Manager® from Aries Systems Corporation

**Unfunded studies**<br>Enter: *The author(s) received no specific funding for this work.*<br><br>**Funded studies**<br>Enter a statement with the following details:<br>• Initials of the authors who received each award<br>• Grant numbers awarded to each author<br>• The full name of each funder<br>• URL of each funder website<br>• Did the sponsors or funders play any role in the study design, data collection and analysis, decision to publish, or preparation of the manuscript?<br>• **NO** - Include this sentence at the end of your statement: *The funders had no role in study design, data collection and analysis, decision to publish, or preparation of the manuscript.*<br>• **YES** - Specify the role(s) played.<br><br>* typeset	
**Competing Interests**<br><br>Use the instructions below to enter a competing interest statement for this submission. On behalf of all authors, disclose any competing interests that could be perceived to bias this work—acknowledging all financial support and any other relevant financial or non-financial competing interests.<br><br>This statement is required for submission and **will appear in the published article** if the submission is accepted. Please make sure it is accurate and that any funding sources listed in your Funding Information later in the submission form are also declared in your Financial Disclosure statement.<br><br>View published research articles from *PLOS ONE* for specific examples.	The authors have declared that no competing interests exist.

**NO authors have competing interests**<br><br>Enter: *The authors have declared that no competing interests exist.*<br><br>**Authors with competing interests**<br><br>Enter competing interest details beginning with this statement:<br><br>*I have read the journal's policy and the authors of this manuscript have the following competing interests: [insert competing interests here]*<br><br>* typeset	
**Ethics Statement**<br><br>Enter an ethics statement for this submission. This statement is required if the study involved:<br><br>• Human participants<br>• Human specimens or tissue<br>• Vertebrate animals or cephalopods<br>• Vertebrate embryos or tissues<br>• Field research<br><br>Write "N/A" if the submission does not require an ethics statement.<br><br>General guidance is provided below. Consult the submission guidelines for detailed instructions. **Make sure that all information entered here is included in the Methods section of the manuscript.**	N/A

**Format for specific study types**<br><br>**Human Subject Research (involving human participants and/or tissue)**<br>• Give the name of the institutional review board or ethics committee that approved the study<br>• Include the approval number and/or a statement indicating approval of this research<br>• Indicate the form of consent obtained (written/oral) or the reason that consent was not obtained (e.g. the data were analyzed anonymously)<br><br>**Animal Research (involving vertebrate animals, embryos or tissues)**<br>• Provide the name of the Institutional Animal Care and Use Committee (IACUC) or other relevant ethics board that reviewed the study protocol, and indicate whether they approved this research or granted a formal waiver of ethical approval<br>• Include an approval number if one was obtained<br>• If the study involved *non-human primates*, add *additional details* about animal welfare and steps taken to ameliorate suffering<br>• If anesthesia, euthanasia, or any kind of animal sacrifice is part of the study, include briefly which substances and/or methods were applied<br><br>**Field Research**<br><br>Include the following details if this study involves the collection of plant, animal, or other materials from a natural setting:<br>• Field permit number<br>• Name of the institution or relevant body that granted permission	
**Data Availability**<br><br>Authors are required to make all data underlying the findings described fully available, without restriction, and from the time of publication. PLOS allows rare exceptions to address legal and ethical concerns. See the PLOS Data Policy and FAQ for detailed information.	No - some restrictions will apply

Powered by Editorial Manager® and ProduXion Manager® from Aries Systems Corporation

A Data Availability Statement describing where the data can be found is required at submission. Your answers to this question constitute the Data Availability Statement and **will be published in the article**, if accepted.<br><br>**Important:** Stating 'data available on request from the author' is not sufficient. If your data are only available upon request, select 'No' for the first question and explain your exceptional situation in the text box.<br><br>Do the authors confirm that all data underlying the findings described in their manuscript are fully available without restriction?	
**Describe where the data may be found in full sentences. If you are copying our sample text, replace any instances of XXX with the appropriate details.**<br><br>• If the data are **held or will be held in a public repository**, include URLs, accession numbers or DOIs. If this information will only be available after acceptance, indicate this by ticking the box below. For example: *All XXX files are available from the XXX database (accession number(s) XXX, XXX.*).<br>• If the data are all contained **within the manuscript and/or Supporting Information files**, enter the following: *All relevant data are within the manuscript and its Supporting Information files.*<br>• If neither of these applies but you are able to provide **details of access elsewhere**, with or without limitations, please do so. For example:<br><br>*Data cannot be shared publicly because of [XXX]. Data are available from the XXX Institutional Data Access / Ethics Committee (contact via XXX) for researchers who meet the criteria for access to confidential data.*<br><br>*The data underlying the results presented in the study are available from (include the name of the third party*	Data cannot be shared publicly because of size of raw data which required confidentiality agreement to require. Aggregate level data are available from the IDMC and UNHCR Institutional Data Access.

Powered by Editorial Manager® and ProduXion Manager® from Aries Systems Corporation

and contact information or URL).<br>• This text is appropriate if the data are owned by a third party and authors do not have permission to share the data.<br><br>* typeset	
Additional data availability information:	

Powered by Editorial Manager® and ProduXion Manager® from Aries Systems Corporation

Manuscript

Click here to access/download;Manuscript;Climate-induced Displacement in Somalia.docx

Deciphering Climate-induced Displacement in Somalia:

A Remote Sensing Perspective

Rahman Momeni[1], Tuba Bircan*[2] and Robert King[1]

[1] *GMV NSK, UK*

[2] *Dept. of Sociology, Vrije Universiteit Brussel, Belgium*

**Corresponding Author:* *tuba.bircan@vub.be*

Abstract

Rapid changes in climate have reflections on all different aspects of our lives, one prominent effect is detreating the living conditions vastly in particular areas where people have no choice but flee. Despite the acknowledgement of this topic, the dynamic of the relationship between the environmental factors and the human mobility is yet to be studied in detail. This study aims to investigate the use of advanced remote sensing analytics for developing advanced climate indicators developed on a micro (district) level using and to study the relationship between climate factors and internally displaced persons. After elaborating on our data sources and the analytics used for indicator development, we discuss different types of events and their aftermath. Our findings confirm that slow-onset climate events and rapid-onset climate events have a different impact on society and the responses depend on the urge due to the deterring aftermath of the extreme weather event and most importantly the capabilities of people. We also emphasise that data quality and availability for the socio-economic indicators are of importance to enhance future studies given the interlinked associations between climate change, economic deprivation, and violent conflict.

Keywords: Remote Sensing, Satellite Data, Human Migration, Internal Displacement

Introduction

Over the past decade, we have witnessed numerous shocks across the globe - be they political, economic, societal or environmental - precipitating humanitarian crises and prompting millions of people to flee or be displaced. The political instability in the Middle East and economic downturns in Latin America since 2015, for instance, dominated international discourse as

mass displacement ensued, with individuals first seeking refuge in neighbouring countries before looking further afield [1-5]. Such crises, and the subsequent emigration they prompt, have been well-documented and rigorously studied.

As of February 2022, the world's gaze has turned towards Ukraine as it faces its own struggle with displacement. While these sudden - albeit not unforeseen - events typically attract substantial attention due to the immediate response required from host countries, a multitude of nations, especially those in Asia and Africa, have been grappling with the severe impacts of chronic or acute environmental disasters for years. When compounded with enduring challenges such as poverty, famine, armed violence, and political instability, the societal effects of these environmental factors intensify, compelling people to relocate. As a result, these individuals first experience displacement within their own countries before ultimately seeking refuge elsewhere.

Indeed, the global population of internally displaced persons (IDPs) has steadily increased, with the 2021 Internal Displacement Index Report indicating that by the end of 2020, the number of IDPs worldwide had surpassed 55 million, double the number of refugees [6]. By developing a nuanced understanding of the root causes behind irregular migration and forced displacement, we can better predict patterns of human mobility. More importantly, we can enhance protection measures for migrants and refugees, providing them with the safety and support they need in these challenging circumstances.

The field of climate-induced migration has seen remarkable advances in contemporary predictions compared to earlier efforts, catalysed by growing interest in studying environmental dynamics and mobility decisions [7-12]. Leveraging innovative data sources and cutting-edge analytics has enormous potential for data collection, consequently augmenting our understanding of human mobility [13,14]. This enhanced knowledge can be

critical in the development of predictive models [15,16], understanding slow-onset processes of climate change [17], and advancing socio-economic indicator development [18].

This paper's primary objective is to scrutinise the relationship between climatic factors and IDPs within the context of Somalia from 2016 to 2019. Our approach employs advanced remote sensing analytics to construct advanced climate indicators at a granular, district level.

The structure of this article unfolds as follows: We begin with a detailed examination of the case of Somalia, elucidating both the environmental conditions and the historical narrative of migration. Subsequently, we delineate our data sources and the methodological framework deployed for creating climate indicators. In the section analysing our results, we discuss our investigation and visualise the indicators through maps to elucidate the relationship between environmental factors and IDPs. In the conclusion, we summarise our findings and offer insights and recommendations aimed at enhancing current practices in this research domain.

Environmental Change and Displacement in Somalia

Our study concentrates on Somalia due to its susceptibility to extreme climatic events, which have triggered considerable internal migration. The nation's fragility and vulnerability stem from a combination of factors including enduring conflicts, protracted epidemic outbreaks, erratic climatic conditions and shocks adversely affecting agriculture, livestock, and rural livelihoods, which are fundamental for food security. Simultaneously, weak social protection systems further exacerbate the situation. The World Bank classifies Somalia as one of the world's most impoverished countries, with approximately 70% of its population living below the poverty line, subsisting on less than $2 per day. Furthermore, the United Nations High Commissioner for Refugees (UNHCR) places Somalia seventh globally in terms of displaced populations.

Since 1990, Somalia has borne the brunt of over 30 extreme weather events, encompassing 12 droughts and 19 floods, precipitating two significant famines in 1991-92 and 2011. These disasters led to more than 3.2 million new displacements, implying the number of movements rather than the number of individuals displaced. Given that over 80% of Somalia's territory features arid and semi-arid climates, and rainfall exhibits high spatial and temporal variability, these adverse environmental impacts and inadequate natural resource management detrimentally affect the nation's economy. Approximately 70% of Somalis are reliant on climate-sensitive agricultural and pastoral activities.

The recurring interplay of drought and floods undermines food security and access to basic services, intensifying vulnerability and prompting displacement at varying scales. UNHCR data reveals that since 2016, over three million Somalis have been displaced due to recurrent extreme droughts and floods. Notwithstanding assumptions concerning "environmental migration" that posit immediate displacement following weather-related disasters such as floods, storms, and drought, the displacement process entails diverse responses and trajectories influenced by contextual conditions and individual capabilities rather than purely rational choices. In regions like Somalia, prone to recurrent extreme weather events, disasters and detrimental environmental conditions may precipitate multiple instances of displacement, further amplifying vulnerability.

Environmental factors, progressively worsening as the climate becomes hotter and drier, directly impinge upon crop, livestock, and forestry-based livelihood systems. Regular and predictable rainfall is critical for the pastoral and rural communities across Somalia. Beyond the immediate effects of abrupt extreme weather events, the slow onset of environmental degradation is a crucial factor provoking displacement towards more habitable regions. These rapid and slow-onset environmental conditions may play divergent roles in individuals' decisions to migrate either internally or internationally at different times. Numerous qualitative

and mixed-methods studies have shown that in many instances, the decision to migrate often materialises as a "last resort" in response to an extreme event, rendering existing living conditions unbearable [19-21]. To distinguish between new displacements and IDPs, Figure 1 denotes the number of movements, not individuals, as individuals can be displaced multiple times, a factor not always reflected in data collected by the Internal Displacement Monitoring Centre (IDMC).

[Figure 1 here]

Figure 2a presents the population density across Somalia, using a granular 100 × 100-meter grid square. Detailed views are shown around (a) Hargeisa, (b) Bosaso, and (c) Mogadishu. In Figure 2b, a land cover map of the region highlights the two primary rivers in Somalia: Shebelle, spanning the central areas, and Juba, crossing the southern regions of the country. The land cover of Somalia, as depicted by the European Space Agency (ESA) under the Africa land cover project in 2016, is represented in Figure 2c. Finally, Figure 2d illustrates the proportion of environmentally driven IDPs between 2016 and 2019 at the regional level. These aggregate figures provide a macroscopic overview of Somalia's severe and erratic climatic conditions and the resultant human responses, often marked by difficult decisions to abandon their livelihoods. However, these annual figures inadequately elucidate the swiftly shifting yet gradually evolving environmental dynamics and their indirect and direct impacts on human lives. Migration decisions can manifest both as an immediate reaction to disasters or a longer-term choice stemming from recurring environmental degradation and bleak long-term prospects. Given the diversity in individuals' responses to similar circumstances, dictated by their physical and economic limitations and their perception of short to long-term risks, it is challenging to discern the degree to which these movements contribute to the continuum of

displacement. Thus, more time-sensitive environmental indicators can provide valuable insights into the relationship between climate alterations and internal displacement.

[Figure 2 here]

The scope of our study, delineated by the bold-blue line in Figure 2d, encompasses regions significantly affected by adverse climatic conditions, resulting in internal displacement. Criteria for area selection involved considerations of population density, proximity to major rivers, and the degree of reported internal displacement attributable to environmental factors. Central and Southern Somalia were selected for this study, as these regions are not only densely populated (Figure 2a) but are also bisected by the Shebelle and Juba rivers (Figure 2b). This geographic configuration provides conducive conditions for crop cultivation and forage production (Figure 2c). Furthermore, these regions have consistently confronted internal displacement due to extreme environmental events (Figure 2d).

Figure 3 delineates the study area at the district level, with a total of 16 districts selected from the Central and Southern parts of Somalia, covering an area of 111,578 km^2. Detailed descriptions of the selected districts are provided in Table 1.

[Figure 3 here]

Table 1. List of districts used as areas of interest (AOI) for agricultural drought indicator

**NO**	**Districts**	**District Area (km^2)**	**NO**	**Districts**	**District Area (km^2)**
1	Saakow	6774	9	Bulo Burto	16054
2	Jowhar	4637	10	Baardheere	15275

3	Balcad	4238	11	Jalalaqsi	3123
4	Jalalqsi	3123	12	Kurtunwaarey	2546
5	Luuq	8260	13	Qansax Dheere	3267
6	Belet Weyne	14867	14	Rab Dhuure	3309
7	Bulo Burto	16054	15	Tayeeglow	6596
8	Garbahaarey	8318	16	Doolow	1618

Data

Our objective is to scrutinise the patterns of environmentally induced internal displacement in Somalia by analysing our derived indicators to discern any relationships with IDPs throughout the Area of Interest (AOI). We aim to understand the connection between extreme weather events, slow-onset climate change, and internal displacement in Somalia between 2016 and 2019. This study spans from January 1, 2016, to December 31, 2019. The start year 2016 was chosen due to the availability of data from the Sentinel-1 (the first satellite in the Copernicus Programme launched by the European Space Agency) and Sentinel-2, an Earth observation mission from the Copernicus Programme that systematically acquires optical imagery at high spatial resolution over land and coastal waters, which was utilised to generate flood and drought maps. Moreover, migration data has been available since 2015.

Previous studies that employ a quantitative empirical approach to the climate-migration nexus can be divided into micro studies, which largely focus on individual and household migration [21], and macro studies, where researchers examine environmentally-induced migration at regional or national levels [9]. In terms of different hazard types, many studies focus on factors associated with climate change, such as changes in precipitation and temperature levels and variability [12]. This paper represents one of the first attempts to analyse climate/environment-induced displacement at the district level, employing environmental indicators derived from daily, weekly, and monthly remote sensing/satellite data.

Remote Sensing Data:

Sentinel-1

~~The~~ Sentinel-1 (S-1) is an open-source Synthetic Aperture Radar (SAR) imaging satellite data with medium spatial resolution as part of the European Space Agency Copernicus programme. ~~The~~ S-1 consists of two satellites launched on 3 April 2014 and 22 April 2016. The SAR system operates within C-band (5.407 GHz) frequencies in one of four acquisition modes and on land the default mode is Interferometric Wide Swath. In total, 32 S-1 GRD scenes (16 pairs) were downloaded from the Alaska Satellite Facility (ASF) and processed spanning from 07/01/2016 to 30/10/2019. The S-1 satellite data were used to map the flood events in Somalia. Figure 4 shows S-1 acquired on 30th May 2016 during a flood event affected Beledweyne in which. The low backscatter areas further from the river are covered by sand presenting a similar backscatter to flooded areas where located along the river are represented flooded areas that can be seen as black colour.

[Figure 4 here]

Sentinel-2

The Sentinel-2 (S-2) dataset is an open-source optical with medium to high spatial resolution satellite data provided as a part of the ESA Copernicus programme. The S-2 mission consists of two satellites: Sentinel-2A and Sentinel-2B. S-2 provides multi-spectral data with 13 bands in the visible, near-infrared, and short-wave infrared parts of the spectrum. ~~The~~ S-2 is equipped with the state-of-the-art Multispectral Instrument, offering large swaths of high geometrical and spectral performance with imagery available at the spatial resolutions of 10 m, 20 m and 60 m. S-2 is broken down into 100 km x 100 km side tiles, called “granules”. In total, 15 S-2

granules were selected which cover the 16 AOI districts. Figure 5 shows the distribution of S-2 granules over the study area.

[Figure 5 here]

On Average, 240 S-2 images were downloaded for each granule from Google Cloud at the Level-1C processing level for a period of January 1st, 2016, to December 31st, 201, covering an area of 180,757 km^2. In total, 4169 of S-2 granules were downloaded that occupied 2.5 TB of memory. These datasets were pre-processed automatically using Sen2cor (version 2.8) [22] to convert to Level-2A ready for processing. The S-2 datasets were used in several environmental indicators such as spectral vegetation indices, drought, flood, and Land Cover (LC) change. In addition, S-2 was used in flood mapping to visually verify the location and date of the flood events with an independent dataset.

NASADEM

The NASADEM is currently the Digital Elevation Model (DEM) with the highest resolution that is freely available for Somalia. The NASADEM was derived from the re-processing of the Shuttle Radar Topography Mission by using the additional layers: Terra Advanced Spaceborne Thermal and Reflection Radiometer, Global Digital Elevation Model, Version 3 data, Ice, Cloud, and Land Elevation Satellite, Geoscience Laser Altimeter System ground control points of its LiDAR shots to improve surface elevation measurements that led to improved geolocation accuracy. The NASADEM was downloaded automatically from the Land Processes Distributed Active Archive Center [23] in ".hgt" format distributed in 1° x 1° tiles (available for all land between 60° N and 56° S latitude), WGS84 for the year 2000 at approximately 30 m pixel spacing. A total of 45 granules were used to generate a DEM mosaic

covering all the S-1 tiles. Other re-processing improvements include the conversion to geoid reference and the use of GDEMs and Advanced Land Observing Satellite Panchromatic Remote Sensing Instrument for Stereo Mapping AW3D30 DEM, and interpolation for void filling. The NASADEM was used to generate the following ancillary layers: a DEM mask, slope mask, and HAND index mask [24].

Soil Moisture Index (SMI)

Soil moisture data represents the current state-of-the-art satellite-based soil moisture data record production, in line with the “Systematic observation requirements for satellite-based products for climate” defined by Global Climate Observing System. The SMI offers spatial resolution equal to 0.25-degrees and with a 10-day temporal resolution. SMI were acquired for the study area between the period of 1978 and 2019 at the ESA Climate Change Initiative Soil Moisture Climate Copernicus. The soil moisture data processed into SMI anomaly that is used to develop the agricultural drought indicator.

Standardised Precipitation-Evapotranspiration Index (SPEI)

~~The~~ SPEI uses the periodic difference between precipitation and potential evapotranspiration. It represents a simple climatic water balance calculated at different time scales [25]. ~~The~~ SPEI offers near real-time information about drought conditions with a 1-degree spatial resolution and a period between images of a month. While SPEI-1 represent the meteorological factor in a monthly bases, SPEI-3 represents a meteorological factor on a more extended period than SPEI-1. SPEI-3 offers steady behaviour because it is less sensitive to the short variation of climate like heavy and light rain that is more likely to be considered on SPEI-1. SPEI-1 and SPEI-3 were downloaded from the period 1950 to 2019 from Spanish National Research Council [26].

IDP Data

We compiled UNHCR, IDMC, and the World Bank, where district-level statistics are available. The specifics of these derived and collected indicators are detailed in the ensuing section. As part of the non-environmental indicators, we used IDP data (sourced from UNHCR[1]) to study the internal migration of environmental refugees since 2015. These IDP values represent the number of internally displaced people each month and include the cause of migration and the originating and destination districts.

Figure 6 presents a map of displacements taking place within and between district levels. During drought events from 2016 to 2019, Doolow emerged as the most preferred destination district for individuals displaced from their home districts. In total, nine migration movements were reported to Doolow, followed by six migration movements to Luuq. On the contrary, Qansax Dheere had the highest number of departures due to drought events within the same period. Similar displacement patterns are showcased in Figure 6b. Notably, flood-induced displacements predominantly occurred within districts, suggesting that flood events were less likely to prompt individuals to leave their districts.

[Figure 6 here]

Socio-Economic Indicators

Data availability and quality present significant challenges for socio-economic indicators in Africa, particularly in impoverished countries like Somalia [27-29]. This issue is further exacerbated when seeking disaggregated data for smaller administrative units. While

1 https://www.unhcr.org/uk/statistics/unhcrstats/60b638e37/global-trends-forced-displacement-2020.html

international databases developed by various institutions cover diverse domains such as democracy, development, and access to services [30], they mostly provide annual statistics at the country or regional level, with limited availability of data for smaller administrative units. To address this challenge, our first step involved selecting data sources that provided disaggregated socio-economic indicators at the district level on a monthly basis. We evaluated data from the World Bank, the International Organization for Migration (IOM), and the United Nations High Commissioner for Refugees (UNHCR) to assess their quality and completeness for the study period from 2016 to 2019. Among these sources, we found that the most sufficient data related to food prices at the district level on a monthly basis, providing information on the prices of essential commodities such as sorghum, maize, and rice.

It is essential to note that while we acknowledge the limitations, inconsistencies, incompleteness, and scarcity of data, the socio-economic variables were not selected as indicators for migration drivers. Instead, they were utilised to control for the impact of environmental factors on the socio-economic conditions in Somalia. By including these variables, we aimed to contextualise the influence of environmental factors on the broader socio-economic landscape of the region.

Methodological Approach: Developing Environmental Indicators

A series of environmental and non-environmental indicators were considered to understand environmental migration better. The goal of using environmental indicators was to measure the direct impact of environmental events on agriculture, human settlements and public infrastructures, whilst non-environmental indicators such as IDP were used to quantify the human impact. The environmental indicators were considered as casual indicators, whilst non-environmental features measured the effect this causes had on the local population that ultimately leading to migration. Here, methodological approaches were developed for each environmental indicators explain in details.

Agricultural drought indicator (ADI)

The methodology was developed for ADI benefiting from the cause-effect relationship for agricultural drought, whereby a shortage of precipitation leads to a soil moisture deficit, resulting in a reduction of vegetation productivity (see Figure 7). It is based on combining anomalies of precipitation (SPEI-1 and SPEI-3), soil moisture (e.g., SMI) and vegetation stress (e.g., NDVI).

- SPEI-1 and SPEI-3: to identify the precipitation deficit.
- SMI anomaly: to identify soil moisture anomalies.
- NDVI anomaly: characterise the subsequent effect of soil water stress on crops.

[Figure 7 here]

As illustrated in Table2, a deficit of rainfall (watch level) causes a reduction of the soil moisture (alarm level), and a deficit of water in the ground which causes vegetation stress which will result in a reduction of NDVI values (alert level) which finally will have an impact on agriculture production and could lead to food insecurity.

Table 2. ADI Classification scheme with three drought levels

Drought level	Colour	Conditions	Interpretation
**Watch**		SPEI-3 < -1 or SPEI-1 < -2	Low SPEI means precipitation deficit.
**Warning**		SMA < -1 & (SPEI-3 < -1 or SPEI-1 < -2)	Precipitation deficit plus a negative anomaly of soil moisture.
**Alert**		NDVIa < -1 & (SPEI-3 < -1 or SPEI-1 < -2)	Precipitation deficit plus a negative anomaly of vegetation 'greenness'.

Flood

The methodology for flood mapping was developed based on the change detection of backscatter intensity of a pair of flood S-1 imagery, one pre-flood and one post-flood SAR imagery. The S-1 imagery was pre-processed using SeNtinel's Application Platform (SNAP) Toolbox 7.0 to perform these steps: (a) Apply orbit file, (b) GRD Border Noise Removal (border limit: 5000, trim threshold: 1.0), (c) Thermal Noise removal, (d) multi-looking (2 looks in range and 2 in azimuth), (e) speckle filtering (Refined Lee filter which is an enhancement of the Lee filter in order to provide better preservation of edges, linear features, and texture information, 5x5 window), (f) range Doppler terrain correction using SRTM 1arc sec and geo-coding to WGS84/UTM Zone 38N. The VV polarisation was considered to detect flooding as opposed to HH or VH polarisation [31-32].

Next, pixel-based change detection between the pre-flood event at time (t_1) and the post-flood event at time (t_2) is performed using the intensity ratio:

$$R = \frac{I_{t1}(i)}{I_{t2}(i)}$$ **Error! Reference source not found.**

Where "$I_{t1}(i)$" is the backscatter intensity of pixel "i" in image acquired at "t_1", and "$I_{t2}(i)$"is the corresponding intensity pixel in image acquired at time "t_2". The pixel ratio $R(i)$ is a sample of a random process whose Probability Density Function (PDF) for uncorrelated SAR intensity images is [32]:

$$P(R) = \frac{\Gamma(2L)\gamma^{L}R^{L-1}}{\Gamma^{2}(L)(\gamma+R)^{2L}}$$ **Error! Reference source not found.**

Where γ is the true change of the radar cross sections σ_0, and L is the number of looks. The probability of detection P_{de} given a threshold T, L and γ is:

$$P_{de}(R > T) = \int^{\infty} P(R)dR$$ **Error! Reference source not found.**

It is important to notice that P depends on the number of looks L. To illustrate the impact of L on the detector performance let us compute the P_{de} for the detection of a 3 dB change ($\gamma = 0.5$) with $T = 0.4$. For L=4 (Sentinel-1) we have: $P_{de} = 0.58$, for L=50 (Lee filter) $P_{de} = 0.86$. These figures point out the importance of speckle filtering for the ratio detector. In our implementation the threshold T is determined by supervised learning, i.e., by estimating the ratio in a known area of no change, as it will be described in Thresholding section.

Apart from common thresholding approaches were tested (e.g., unimodal thresholding approaches such as T-point and maximum deviation), a change detection approach employed based on a ratio between pre-flood and post-flood imagery. This change detection approach minimises the misclassification of arid land as flooded, due to similar backscatter of desert surfaces and flooded areas in arid areas like Somalia. The thresholds in this study were calculated by supervised inspection of the imagery. The optimal threshold was calculated for a flooded pixel (P_F) and for a non-flooded pixel (P_{NF}). A coefficient f_c was calculated based on

several trial and error tests and also confirmed by findings in [34]. The μ and σ are the mean and standard deviation of the no-change area selected in the ratio image R:

$$P_F \geq (\mu[R] + f_c * \sigma[R]) \qquad P_{NF} < (\mu[R] + f_c\, \sigma[R])$$ **Error! Reference source not found.**

Where P_F is the flooded pixel; P_{NF} is the non-flooded pixel; f_c is a coefficient factor found based on several iterations; μ = mean R and σ = the R standard deviation over the stable area. Next, ancillary masks were generated based on the NASADEM. A threshold was set to generate a binary slope mask. Slopes ≤ 3° were considered to be prone to flooding and assigned a pixel value of 1, while slopes >3° are considered not likely to flood, assigned a pixel value of 0 and were removed from further analysis. This step was essential to remove any pixels that may have shown a change in backscatter due to the angle of signal return from a higher elevation such as hills. Areas presenting elevation ≥ 200 m were also masked and considered unlikely to be flooded. The slope and elevation threshold were selected based on the values used in the literature (e.g., [31, 34]).

Finally, a further terrain filter was applied to remove areas which can be considered unlikely to be flooded based on the Height Above Nearest Drainage (HAND) [24]. A binary HAND mask was generated where pixels with HAND values ≥15 m were considered not flood-prone while pixels with HAND < 15 m were considered likely to be flooded. Post-processing of the resulting S1 flood map was performed to remove isolated clusters of pixels < 50 pixels in size (equivalent to 0.25 ha) reducing the false detections arising from noise. The algorithm removes and replaces pixels below the selected threshold with the pixel value of the largest neighbour polygon. Finally, a morphological binary closing applied to fill holes smaller than the chosen disk radius (*r* = 5) [35].

Land Cover (LC) change

A LC classification scheme was developed to study the impact of the natural disasters like drought and flood on agricultural food productivity and human activity. This motivated the inclusion of the cropland class, containing food producing arable land as well as food producing orchards. Meanwhile the Settlement class was created to include any apparent permanent human structure. The two principles “background” classes in this product where the Grassland/Shrubland class and the Soil/Sparse vegetation class. The primary differentiator between these two classes is the quantity of vegetation present, with Grassland/Shrubland dominated by low bushes, and occasional thick grass coverage, whilst the Soil class contains minimal vegetation. The study area is covered mostly with grasslands and shrubland whilst the majority of the croplands, forest, and settlement regions are distributed along the Shebelle River. The classification scheme can be seen below in Table 3:

Table 3. Classification scheme of AOI land cover with six members

ID	Class	Colour	Description
**0**	Agricultural Fields		Arable land used for crop growing. Food producing orchards are also including in this class.
**1**	Forest		Area covered by dense tree coverage, such that the underlying ground coverage cannot be seen.
**2**	Grassland / Shrubland		Open areas covered by dense non-arable vegetation including predominately from grass and bushes. Class may also include sparse tree coverage.
**3**	Settlement		Region forming a permanent human settlement.

**4**	Soil / Sparse vegetation		Open area covered by bare soil, sand and low-density vegetation.
**5**	Water		Water bodies including lakes, rivers, ponds, and canals. Also includes regions of flooding covered by ground water.

The entire of four spectral bands of S-2 with 10m resolution (blue, green, red, near infrared) and two 20m resolution (Short Wave Infrared 1 and 2) were used at the Level-2A in the training of the model as well as for its evaluation. In addition, the NDVI was used to improve distinguishing the cropland and forest classes.

A U-Net based model was used as the model architecture for this product, with results from the preliminary pilot studying showing this architecture giving the strongest results both in terms of validation metrics and visual results. The U-Net architecture is a type of Convolutional Neural Network (CNN) containing layers which "slide" multiple trained filters over the image creating several output channels. In a U-Net all convolutions have a kernel size of 3x3, meaning that a pixel and its immediate neighbours are all that are selected.

The encoding portion of the network also used skip connection to form so called ResBlocks, which have the advantage of allowing the input image to propagate deeper through this portion of the network and thus prevent issues such as vanishing gradients from being prominent. The loss function used in the training of this network was a weighted average of the cross entropy and dice loss between the two images. The cross entropy is a very commonly used loss function for classification tasks, it works by calculating the information gain using the predicted image as the prior and the true image as the posterior. The two components of the loss function were calculated separately with the dice cross comprising 90% of the loss function and the cross entropy the remaining 10%.

The network was trained using the Adam Optimizer combined with a learning rate scheduler. The scheduler reduces the learning rate gradually throughout training by a constant factor of 0.92 per epoch. In total, the network was trained for 100 epochs. The U-Net model had various hyperparameters which affected the details of the network architecture and training parameters that needed to be tuned in order to get an optimal result. There were two main hyperparameters that defined the structure of the U-Net, its depth, and its starting features. The depth of the U-Net is defined as the number of skip connections between convolutional encoding/decoding blocks. The depth of the model is 5, as there are 5 total skip connections between encoder/decoder blocks. The other key hyperparameter in the structure of the network was the number of starting channels that were used. A depth of 5 was shown to give the highest accuracy and F1 score on the validation set and was able to fit in the GPU memory. For the number of starting channels, as value of 64 was chosen by the same process as before of experimenting with values from 16 to 128, with 64 giving the highest accuracy on the validation.

A series of polygons were created for training and testing the U-Net model using S-2 imagery. These set of polygons selected during the two annual growing seasons and the polygons were free from. The U-Net model trained on 128x128 images containing the seven input channels. The first approach of gathering training samples, inspired by an approach [36], synthetic training images were created from the training polygons. The algorithm to generate the synthetic training images via tessellation worked by randomly selecting a polygon from the training polygon set and then rasterizing it which involved calculating all input features. Each rasterised polygon was sampled randomly weighted by its area, meaning that larger polygons were more likely to be selected than smaller ones. In total of 1024 128x128 training images as well as 128 128x128 testing images.

The second approach to generating training images for the U-Net was to select a square ground region and attempt to label each pixel in the image using the S-2 data as a reference. Each pixel in an image was hand labelled into one of the six classes defined in the classification schema. After augmentation this led to a total of 985 ground truth images of 128x128 in size, generation in total 2009 training images from both methods available for training. These polygons were then divided with 70% of the polygons being used for training and 30% for testing.

The LC Change was calculated based detection of changes between two LC maps: a before and after map. The LC change between the two maps can then be calculated by doing a simple mapping of the form:

$$LCChange = LC_{after} + LC_{before} * \# classes$$ **Error! Reference source not found.**

The number of classes was 6 resulting to a total of 36 possible LC Change classes representing each possible transition of any class to any other class (including conservation). In the case where one or both LC values were unavailable for a given pixel due to the cloud mask, then the LC Change at this pixel was also masked. All possible transitions are seen in Table 4.

Table 4. Demonstration of the mapping of the transition between two Land Cover classes to an integer value that was stored in the Land Cover Change raster file.

To / From	Agricultural Field	Forest	Grass/ Shrub	Settlement	Soil	Water
**Agricultural Field**	0	1	2	3	4	5
**Forest**	6	7	8	9	10	11

**Grass/Shrub**	12	13	14	15	16	17
**Settlement**	18	19	20	21	22	23
**Soil**	24	25	26	27	28	29
**Water**	30	31	32	33	34	35

Analysis and Results

Drought and Floods

In total, 48 ADI mosaic produced monthly with 20 m spatial resolution in GeoTIFF format, which is a public domain metadata standard which has the georeferencing information embedded within the image file, such as maps. Figure 8 shows an example of drought map of August 2017, where the AOI was affected by moderate and severe drought), primarily in the south of the AOI at the lower Shebelle River.

[Figure 8 here]

River floods are the most common and one of the main causes of environmental migration in Somalia, which happens when water overflows a riverbank due to a prolonged period of rainfall. The flood algorithm was developed based on change detection technique using Sentinel-1 data from before and after a flood. In addition, the Digital Elevation Model was used to calculate the slope of the study. Whilst the drought maps were produced monthly, flood maps were produced based on flood events. **Error! Reference source not found.** shows the overall flood mosaic map, where in 2016, 865.5 km^2 of the AOI was flooded, with the Belet Weyne, Saakow and Jowhar districts mostly affected (Figure 9a). In 2017, floods affected the districts of Saakow and Jowhar, covering 2263.4 km^2 (Figure 9b), whilst in 2018 several districts were flooded (including Hiiraan -Figure 7) with an area equal to 3552.1 km^2 (Figure

9c). Lastly, in 2019 the total area flooded was 2657.9 km^2. The flood was used as a base map alongside the population map to estimate the number of people affected by each flood event.

[Figure 9 here]

Land Cover (LC) and LC change maps for drought and flood events

The last environmental indicator was comprised of the Land Cover (LC) and LC change maps. The LC helps assess the impact of natural threat phenomena like floods and drought on agricultural food productions (like orchards and croplands) and human settlements (like towns and villages). First, LC maps were produced to show the Earth's different land cover patterns in five different classes (soil/sparse vegetation, water, agriculture field, forest, and grassland/shrubland).

S-2 Level 2A imagery was the primary data input for LC maps. A state-of-art U-Net based algorithm produced LC maps during drought and flood events. The convolutional nature of U-Nets allows them to incorporate information from neighbouring pixels in the prediction which can lead to a stronger detection of different textural features seen in the base images. LC is a fundamental environmental variable for understanding the causes and trends of human and natural processes. LC refers to the physical material at the earth's surface like grass, soil, impervious surfaces, and trees. The information extracted from the land cover features and their associated changes can help understand the impact of environmental threats on the amount of variation of crops. Accurate identification and extraction of different land cover features (e.g., agricultural fields) is important to assess the distribution across agricultural regions.

As a result, 17 LC maps were produced according to the flood events resulting in significant IDPs from 2016 to 2019. The dates selected to produce the LC maps were selected using the first usable Sentinel-2 image that was available after the date reported for each flood event.

However, the LC maps for drought events (see Figure 10) were generated as an aggregate for growing seasons (Gu and Dayr). Eight aggregate LC maps were produced, providing an overall representation of the Land Cover for each agricultural growing season between 2016 and 2019. The figure AG shows the aggregate LC map on Dayr 2018. The Agricultural field with the light green colour in Figure 10 represents the croplands and orchards mainly distributed across the Shebelle and Juba rivers.

[Figure 10 here]

Next, LC change maps were produced to highlight the changes in agricultural fields and human settlements due to flood and drought events. LC change maps for flood events were generated by comparing the LC maps after flood events to the corresponding aggregate LC maps. For drought, six LC Change maps were produced by comparing each aggregate to the previous years' aggregate map for the identical season; for example, Gu 2016 was compared to Gu 2017. The flood LC Change map in Figure 11 shows the clear impact of floods on the agricultural fields and settlements of the affected areas, whilst the LC Change map for drought showed a significant fraction of agricultural fields in drought areas were destroyed or damaged. All the LC and LC change maps were produced in 10m spatial resolution. **Error! Reference source not found.** 11 demonstrates the impact of the flood by showing the agricultural fields and settlements that were lost in the Beledweyne District in Figures 11c and 11d respectively. Most notably, evidence of migration from major urban areas close to the rivers to smaller communities far away from rivers can be seen after flood events. This suggests that migrants from major floods try to stay close to their homes but outside the direct disaster zone.

[Figure 11 here]

An unsupervised clustering was performed to explore the impact of flood events further. The flood events labelled as regular showed to have similar characteristics. In contrast, extreme floods led to higher IDPs, more affected people, and larger damage to agricultural fields and human settlements. It is expected that, compared to regular floods that are part of the agricultural climate and essential for the growing season, extreme floods are more likely destructive and lead to migration.

Compared to flood events, drought events typically take place over a much longer time scale. As such for determining the LC change, a single post drought image cannot be defined in the same way as for flood. As a result, six drought LC change maps were produced based on comparing the matching seasonal aggregates LC. In addition, the change in the agricultural class was the only component of the drought LC Change, as drought should not directly destroy the settlement class as with flood. **Error! Reference source not found.** shows an example of the LC Change maps that were produced between the 2019 and 2018 Gu season showing the impact of a Drought in April 2019 around the Balcad region.

[Figure 12 here]

Linking Environmental Factors and Internally Displaced People (IDPs)

A series of plots were generated for all districts in our AOI to visually demonstrate the impact of environmental threats like drought and flood on internal displacement in Somalia. The plots of the Qansax Dheere and Doolow districts were compared visually. The Qansax Dheere district had the highest outgoing environment-related displaced people, while Doolow district on the border with Ethiopia had the highest incoming environment-related displaced people.

ADI and loss of agricultural fields

Agricultural fields (in hectares) in this study are important to investigate because they provide sources of food for humans and livestock. In addition, they are the main source of income and survival in rural areas in Somalia. **Error! Reference source not found.** shows the impact of drought on agricultural fields' variation for the Qansax Dheere district from 2016 to 2019. The percentage of the district affected by three different drought levels is shown as pale orange (watch level), orange colour (warning level), and dark brown colour (alert). The purple curve represents the total agricultural fields lost.

[Figure 13 here]

As **Error! Reference source not found.**.a shows, the total agriculture fields started to reduce when the purple curve moves upright, three months after the drought started in Feb 2016. In 2017, the agricultural fields began to disappear as a drought started in May, and despite the drought intensity increasing between May to July, the agricultural fields did not shrink. This smaller reduction in the agricultural fields can be explained by the natural crop production increase which occurs during the agricultural growing season following the Gu growing season seen in **Error! Reference source not found.** 13b. Despite more areas of the Qansax Dheere district being affected by the lowest degree of drought between August and December 2018, the reduction in the agricultural fields was minimised. It can be explained by the importance of the degree of drought that affected agricultural fields. In other words, the agricultural fields are affected mainly by the highest level of drought and are least affected by the warning level of drought. This can be seen in Figure 13d where agricultural fields' coverage started to diminish from June to October 2019 since more areas of the Qansax Dheere district were affected by alert and warning drought. Reviewing plots for other districts repeats the same story: generally speaking, agricultural fields were reduced due to increasing drought severity

and extent. These plots help to validate our ADI metric that we chose by showing a clear link to agricultural devastation thus agreeing with the cause-effect hypothesis proposed. It can be suggested that the ADI particularly in severely affected regions can be indicative of economic devastation, as seen on agricultural fields' reduction.

ADI and displacements

A series of plots were generated for every sixteen districts to visualise the variation on IDPs during the drought events. Here only two IDP plots for Qansax Dheere and Doolow are represented. Qansax Dheere represents the district with the highest outgoing migration events during 2016 and 2019. However, the Doolow district is chosen since it has the highest number of incoming displacements. **Error! Reference source not found.** and **Error! Reference source not found.** provide a better picture of displacement movements due to drought for the entire four years of study controlling for other factors including flood events, growing seasons. The background of each figure shows the percentage of the district (Qansax Dheere, in the case of **Error! Reference source not found.**) affected by watch, warning, and alert on the left vertical axis. Then, you can find the two vertical strips per year representing the Gu and Dayr growing seasons, respectively. It is expected that precipitation will rise by the start of those seasons, consequently decreasing the percentage and intensity of the drought through the growing seasons.

Three different IDP values are presented in the next figures: net IDP, internal IDP, and total IDP. The net IDPs represents the overall movement for a given month factoring those leaving as a result of environmental factors and those migrating to this district to escape devastation from other districts. A negative net IDP means that more people moved in the district rather than leaving. The internal IDP figures reflect only the number of people displaced from their

homes who decided to remain within their home districts, whilst the total IDP numbers show the total number of people who were displaced in some way during in a period of a month. As shown in **Error! Reference source not found.**, the first displacement wave started two months after the drought event started in November 2016 (the start of the dry season) and the majority of the displaced population returned to their homes in Qansax Dheere by May 2017. It took nearly one year for the remaining migrants to return to their homes. The second drought period was between June and September 2017, leading to around 3,000 displaced people. This low number of displacements can be explained by the previous drought during which many people had already left the district. In addition, the drought that occurred during the agricultural growing season suggests there may be enough food available for them to stay. The number of displaced people during the third drought period between May and September 2019 did not exceed 3,000 people. This can be explained by the availability of food during the agricultural growing season, similar to the previous drought period.

[Figure 14 here]

Two major drought periods were identified for the Doolow district between 2016 and 2019 in Figure 15. The first drought started in June 2016 and ended in January 2017, leading to a significant external displacement from Doolow two months later. The second noticeable drought event occurred a month after the first migrants had returned home and lasted until November 2018. Like the previous time, the migrants moved out of Doolow two months after the drought started. This indicates that people might need to collect resources to be able to move, and the decision to move can follow with a time lag needed to realise the “capabilities” [26] [9].

[Figure 15 here]

It may seem that due to higher precipitation during the Gu and Dayr growing seasons, the impact of droughts decreases during these seasons. However, unexpectedly we saw examples against this. In the case of Qansax Dheere the displacements were often external with no incoming migration. However, Doolow had a high negative net IDP meaning that large numbers of people moved into Doolow because of drought in other districts is Somalia. This is not unexpected as people who managed to arrive in Doolow are closer to humanitarian support such as safety, food and healthcare provided at the nearby refugee reception centres and camps. Doolow is one of the main exit points from Somalia to Dolo Ado, a small Ethiopian border town, and mostly serves as is a hub for cross-border flows of displaced people.

Flood and displacements

Compared to drought, the impact of flood is mostly localised and limited to the districts. Figure 16 illustrates that the majority of displacements due to floods remained internal within a district. Because beside internal displacements, Belet Weyne recorded having two external displacements. The flood events representing regular and extreme floods, respectively are shown as two different lines. From the three flood events, only two (April 2018 and November 2019) led to displacements movements in Belet Weyne district.

[Figure 16 here]

The effect of flood events on displacement is much more immediate than that of drought. Internal displacement occurs shortly after the flood followed by a quick return to the IDPs' homes after water receded. Drought on the other hand, is more gradual and people start to leave

their homes with a delay after the drought started. In addition, it takes much longer for those who decide to return home as it is likely that those who are migrating have decided that their previous living conditions were unviable.

To sum up, the impact of droughts on departure and return displacements is more complex than that of floods. However, reviewing the entire AOI period on drought and IDPs for all districts reveals that it is not necessary for a district affected by a severe drought to also result in a high displacement of people.

Discussion

In this research, various environmental indicators were constructed using remote sensing datasets, such as Agricultural Drought Index (ADI), flooding, and Land Cover (LC) change, to illustrate the effects of extreme events on ground conditions in Somalia. The ADI methodology was initially developed based on the cause-effect correlation in agricultural droughts, where insufficient precipitation incurs a soil moisture deficit, leading to a decline in vegetation productivity. A monthly time series of ADI was generated, corresponding to the varying stages of the cause-effect drought, stratified into three severity levels: watch, warning, and alert. The precision of drought monitoring could potentially be enhanced by augmenting data input via in-situ measurements with superior spatial resolution, or by persistently employing remote sensing and model ensemble techniques.

In addition, a sequence of flood maps was produced utilizing a change detection and thresholding approach, considering pre- and post-flood Sentinel-1 imagery closely aligned with the flood event. Data availability constraints were especially prominent prior to the launch of Sentinel-1B. One of the encountered challenges during this research was the difficulty in locating optimal satellite data, such as Sentinel-1 images close to pre- and post-flood dates, and

historical Sentinel-2 data prior to 2015. Furthermore, a deficit in ground truth data for direct validation of the ADI and LC change map was observed, presenting a significant limitation to the study.

Finally, a series of Land Cover (LC) change maps was developed to evaluate the repercussions of natural hazard events, specifically drought and flood, on various land cover types, particularly those associated with agricultural productivity and human settlements. The LC methodology was devised based on the state-of-the-art U-Net technique, which was trained on the Sentinel-2 Level-2A dataset. The convolutional characteristic of U-Nets enables the assimilation of information from surrounding pixels into predictions, offering superior detection of large-scale structures within images compared to the single-pixel detection of Random Forests.

By collecting training data via polygons, the U-Net training process was substantially expedited compared to the laborious task of manually labelling pixels across an entire image. Consequently, the final trained model surpassed conventional machine learning techniques typically employed for land cover purposes, such as Random Forest. However, a noteworthy limitation of LC change pertains to the inherent difficulty in disentangling the impact of drought from concurrent events during the entire growing season. These concurrent events may include flooding and deliberate shifts in cultivation patterns, which could convolute the interpretation of the results.

In examining the environmental perspective, our analysis identified 2017 as the driest year for Somalia, while 2018 and 2019 were heavily impacted by floods. Periods of routine rainfall, namely the Gu and Dayer seasons, are characterised by increased precipitation, which tends to mitigate drought impact during these growing seasons. In regions such as northern and central Somalia, where rapid-onset and slow-onset extreme climatic events occur either sequentially

or concurrently, categorizing environmental factors as beneficial or detrimental is complex. This complexity arises, for instance, when considering the ameliorating effect of floods following severe droughts in agricultural areas. However, such environmental fluctuations may not invariably manifest as advantageous impacts on livelihoods.

As we investigate how these extreme weather phenomena affect the day-to-day lives of individuals, it becomes apparent that the consequences of rapid-onset events (such as floods) and slow-onset events (like droughts) diverge, particularly regarding individual or household response times. For instance, flood events prompt swift displacement, with minimal response time, indicating that people initiate evacuation even shortly before floods occur, and often return to their homes soon after the floodwaters recede, contingent upon the area's recovery. Our study confirms that the effects of floods are predominantly localised and confined to districts, and most flood-induced displacement results in internal displacement within the same districts. Conversely, drought is a more progressive phenomenon, making adaptation to these more drastic changes challenging. Displacement due to drought commences with a delay and subsequently sees a gradual increase in the number of IDPs. Additionally, the reversion to habitual residences post-drought is a considerably prolonged process.

As we scrutunise the underlying causes of displacement, we identify a diverse and intersectional assortment of reasons encompassing climate-related threats, conflict, extreme poverty, and food insecurity. Given the profoundly inter-correlated nature of these factors and events, evaluating their marginal effects proves unfeasible. When individuals encounter the repercussions of these threatening conditions on their livelihoods, well-being, and security, whether in the short, medium, or long term, the decision to relocate can be construed as a strategic response. However, the predictability of events in terms of timing and the magnitude

of their impact varies, thus individuals may opt for immediate reactions to avoid imminent starvation and material deprivation, rather than employing strategic responses.

Our research has shown that, at present, responses largely remain at an individual level, particularly as mobility in reaction to slow-onset events significantly hinges on individual "capabilities" [37]. Considering projections of climate change of similar or greater magnitude, it appears unattainable to develop a robust assessment of current and future impacts solely through macro analyses of aggregate socio-economic statistics of vulnerable regions and existing climate products. Specifically, segregating the intertwined facets of economy, security, and climate appears to be an insurmountable challenge.

Climate change and extreme weather events not only instigate migratory movements but also precipitate other factors such as economic deprivation and food insecurity. In the context of Somalia, a country that has been enduring both environmental hazards and armed conflict for decades, the urgent need for durable solutions to its humanitarian crisis is inescapable. That being said, the resources required to effectively address internal displacement and migration in Somalia (and similarly afflicted countries) will extend beyond the capacity of Somalia itself and necessitate multinational and multilateral collaboration, as the principal long-term strategy should aim at minimizing the factors exacerbating climate change.

Considering climate-induced migration, the task of delineating the intricate and multifaceted relationship between environmental variables and human migration extends beyond mere conceptual discourse. An increasing body of research points towards the inadequacy of regarding climate factors as isolated and peripheral drivers of human mobility; a more nuanced comprehension is imperative to decipher the interactive dynamics intrinsic to this nexus (i.e., [9, 11]). Especially in a context like Somalia, which is susceptible to severe climatic conditions and persistent conflict, the fundamental causes of displacement prove challenging to explicate due to the relentless interconnections between conflict and environmental degradation.

In encapsulating the technical limitations of this study, our most significant hurdle was procuring a dataset—whether satellite or ground data—that aligns with the timescale and resolution of our AOI. For instance, in generating the flood product map, an image from Sentinel-1 soon after the flood event is essential for capturing the extent of the flooding. Nevertheless, the prolonged revisit time of the Sentinel-1 sensor sometimes resulted in an unavailable image of the flooded region until several days post-flood commencement. Consequently, this could lead to an inaccurate representation of the flood's full scope.

Moreover, for Land Cover products, the caliber of ground truth data is crucial as it directly influences the quality of the predictions. This includes a dearth of ground truth data for validating past drought occurrences. While datasets like the Land Cover Net exist, they are typically too expansive, encompassing the entire African continent, or they possess an unspecific classification scheme that doesn't correspond with ours.

For other products such as drought, our only recourse was validating the predicted drought regions through reliable drought reports from local sources. Future research on drought mapping should concentrate on better portraying the drought status, either by enhancing data input through the use of in-situ measurements with a finer spatial resolution, or by persisting with remote sensing and model ensemble techniques.

Adding to the challenges of this study, we faced limitations in accessing high-quality, micro, longitudinal socio-economic data, particularly on a granular level such as district-based data. The issue of data quality and availability remains a significant challenge, especially for economically disadvantaged African nations [28-29].

A key contributing factor in our case study of Somalia is the longstanding armed conflict and war the country has been experiencing for decades. This has resulted in a lack of governmental reports due to the inherent difficulties in collecting ground data, with only limited information available from international observers.

Despite these challenges, our study offers an innovative evaluation of detailed environmental indicators and IDPs in Somalia. This approach could potentially be expanded for more extended time periods and broader geographical coverage. Future research could examine cross-border mobility and environmental factors. However, given our findings and existing data challenges, we assert that one of the first steps should be improving data quality and availability at both the national and international levels for regions most affected by climate change.

Finally, we propose that triangulating quantitative and qualitative information can aid in illuminating such complex phenomena. This approach could support future research aimed at enhancing our understanding of climate-induced migration.

Acknowledgement

This research is supported by the European Commission through the Horizon2020 European project: "HumMingBird – Enhanced migration measures from a multidimensional perspective" (GA: 870661).

References

[1] Popa V, Cocos O. Syria, an agonizing country. The socio-economic, political and environmental implications of the conflict. Romanian Review on Political Geography/Revista Române Geografie Politica, 2015 May;17(1):29-37.

[2] Aburas R, Najeeb A, Baageel L, Mackey TK. The Syrian conflict: a case study of the challenges and acute need for medical humanitarian operations for women and children internally displaced persons. BMC medicine. 2018 Dec; 16(1): 1-6.

[3] Huynh BQ, Basu S. Forecasting internally displaced population migration patterns in Syria and Yemen. Disaster medicine and public health preparedness. 2020 Jun; 14(3): 302-7.

[4] Podesta J. The climate crisis, migration, and refugees. Who's Certified [Internet]. 2018. Brookings Institute. [cited 2022 Sep 5]. Available from: https://www.brookings.edu/research/the-climate-crisis-migration-and-refugees.

[5] Roth BJ. Temporary Shelter: Venezuelan Migrants and the Uncertainty of Waiting in Colombia. Journal of Immigrant & Refugee Studies. 2021 Aug 28: 1-3.

[6] IDMC. Global Report on Internal Displacement 2021. Who's Certified [Internet]. IDMC. 2022. [cited 2023 Mar 8]. Available from: https://www.internal-displacement.org/global-report/grid2021/

[7] Laczko F, Aghazarm C. Migration, Environment and Climate Change: assessing the evidence. International Organization for Migration (IOM); 2009.

[8] Piguet E, Pécoud A, De Guchteneire P. Migration and climate change: An overview. Refugee Survey Quarterly. 2011 Sep 1; 30(3): 1-23.

[9] Abel GJ, Brottrager M, Cuaresma JC, Muttarak R. Climate, conflict and forced migration. Global environmental change. 2019 Jan 1; 54: 239-49.

[10] Boas I, Farbotko C, Adams H, Sterly H, Bush S, Van der Geest K, Wiegel H, Ashraf H, Baldwin A, Bettini G, Blondin S. Climate migration myths. Nature Climate Change. 2019 Dec; 9(12): 901-3.

[11] DeFries RS, Edenhofer O, Halliday AN, Heal GM, Lenton T, Puma M, Rising J, Rockström J, Ruane A, Schellnhuber HJ, Stainforth D. The missing economic risks in assessments of climate change impacts.

[12] Hoffmann R, Šedová B, Vinke K. Improving the evidence base: A methodological review of the quantitative climate migration literature. Global Environmental Change. 2021 Nov 1; 71: 102367.

[13] Sîrbu A, Andrienko G, Andrienko N, Boldrini C, Conti M, Giannotti F, Guidotti R, Bertoli S, Kim J, Muntean CI, Pappalardo L. Human migration: the big data perspective. International Journal of Data Science and Analytics. 2021 May; 11(4): 341-60.

[14] Momeni R, Bircan T, King R. Environment-induced internal displacement. The case of Somalia (Deliverable 4.3). 2022. Leuven: HumMingBird project 870661 – H2020.

[15] Monteiro J, Martins B, Pires JM. A hybrid approach for the spatial disaggregation of socio-economic indicators. International Journal of Data Science and Analytics. 2018 Mar; 5(2): 189-211.

[16] UNFPA S. Population Survey of Federal Republic of Somalia for the 18 Pre-War Regions of Somalia. Nairobi: UBON, Publishing Service Section, Nairobi. 2014.

[17] Achour ME, Lacan NI. Drought in Somalia: a migration crisis. The State of Environmental Migration. 2012; 6:12.

[18] Jayawardhan S. Vulnerability and climate change induced human displacement. Consilience. 2017 Jan 1(17): 103-42.

[19] Lindvall K, Kinsman J, Abraha A, Dalmar A, Abdullahi MF, Godefay H, Lerenten Thomas L, Mohamoud MO, Mohamud BK, Musumba J, Schumann B. Health status and health care needs of drought-related migrants in the Horn of Africa—a qualitative investigation. International journal of environmental research and public health. 2020 Aug; 17(16): 5917.

[20] Van Baalen S, Mobjörk M. Climate change and violent conflict in East Africa: Integrating qualitative and quantitative research to probe the mechanisms. International Studies Review. 2018 Dec 1; 20(4): 547-75.

[21] Warner K, Afifi T. Where the rain falls: Evidence from 8 countries on how vulnerable households use migration to manage the risk of rainfall variability and food insecurity. Climate and Development. 2014 Jan 2; 6(1): 1-7.

[22] European Space Agency (ESA). ‘Sen2cor’ [dataset]. Who's Certified [Internet]. European Space Agency. 2020 - [cited 2023 Feb 20]. Available from: https://step.esa.int/main/third-party-plugins-2/sen2cor/

[23] NASA. NASADEM_HGT v001 [dataset]. NASA. Who's Certified [Internet]. 2000. NASA. [cited 2022 Nov 14]. Available from: https://lpdaac.usgs.gov/products/nasadem_hgtv001/

[24] Rennó, C. D. et al. (2008). HAND, a new terrain descriptor using SRTM-DEM: Mapping terra-firme rainforest environments in Amazonia. *Remote Sensing of Environment*, 112(9), 3469–3481. doi: 10.1016/j.rse.2008.03.018.

[25] Beguería S, Latorre B, Reig F, Vicente-Serrano S. SPEI Global Drought Monitor, CSIC [data set]. Who's Certified [Internet]. Evanston (IL): The American Board of Medical Specialists. 2000 - [cited 2022 Mar 10]. Available from: https://spei.csic.es/map/maps.html#months=1#month=6#year=2020

[26] Cammalleri C, Arias-Muñoz C, Barbosa P, de Jager A, Magni D, Masante D, Mazzeschi M, McCormick N, Naumann G, Spinoni J, Vogt J. A revision of the Combined Drought Indicator (CDI) as part of the European Drought Observatory (EDO). Natural Hazards and Earth System Sciences Discussions. 2020 Aug 7; 2020: 1-34.

[27] Devarajan S. Africa's statistical tragedy. Review of Income and Wealth. 2013 Oct; 59: S9-15.

[28] Hoogeveen J, Nguyen NT. Statistics reform in Africa: aligning incentives with results. The Journal of Development Studies. 2019 Apr 3; 55(4): 702-19.

[29] Naudé W, Vinuesa R. Data deprivations, data gaps and digital divides: Lessons from the COVID-19 pandemic. Big Data & Society. 2021 Aug ;8(2): 20539517211025545.

[30] Bédécarrats F, Cling JP, Roubaud F. The data revolution and statistical challenges in Africa: Introduction to the special report. Afrique contemporaine. 2016; 258(2): 9-23.

[31] Clement MA, Kilsby CG, Moore P. Multi- temporal synthetic aperture radar flood mapping using change detection. Journal of Flood Risk Management. 2018 Jun; 1(2): 152-68

[32] Twele A, Cao W, Plank S, Martinis S. Sentinel-1-based flood mapping: a fully automated processing chain. International Journal of Remote Sensing. 2016 Jul 2; 37(13): 2990-3004.

[33] Oliver, C. and Quegan, S. (2004) *Understanding Synthetic Aperture Radar Images*. SciTech Publishing.

[34] Long S, Fatoyinbo TE, Policelli F. Flood extent mapping for Namibia using change detection and thresholding with SAR. Environmental Research Letters. 2014 Mar 11; 9(3): 035002

[35] van der Walt S, Schönberger J, Nunez-Iglesias J, Boulogne F, Warner J, Yager N, Gouillart E, Yu T. t contributors (2014). scikit-image: Image processing in python.

[36] Laban N, Abdellatif B, Ebeid HM, Shedeed HA, Tolba MF. Sparse Pixel Training of Convolutional Neural Networks for Land Cover Classification. IEEE Access. 2021 Mar 30; 9: 52067-78.

[37] De Haas H. A theory of migration: the aspirations-capabilities framework. Comparative Migration Studies. 2021 Dec; 9(1): 1-35.

Figure 1

Click here to access/download;Figure;Fig1.tiff

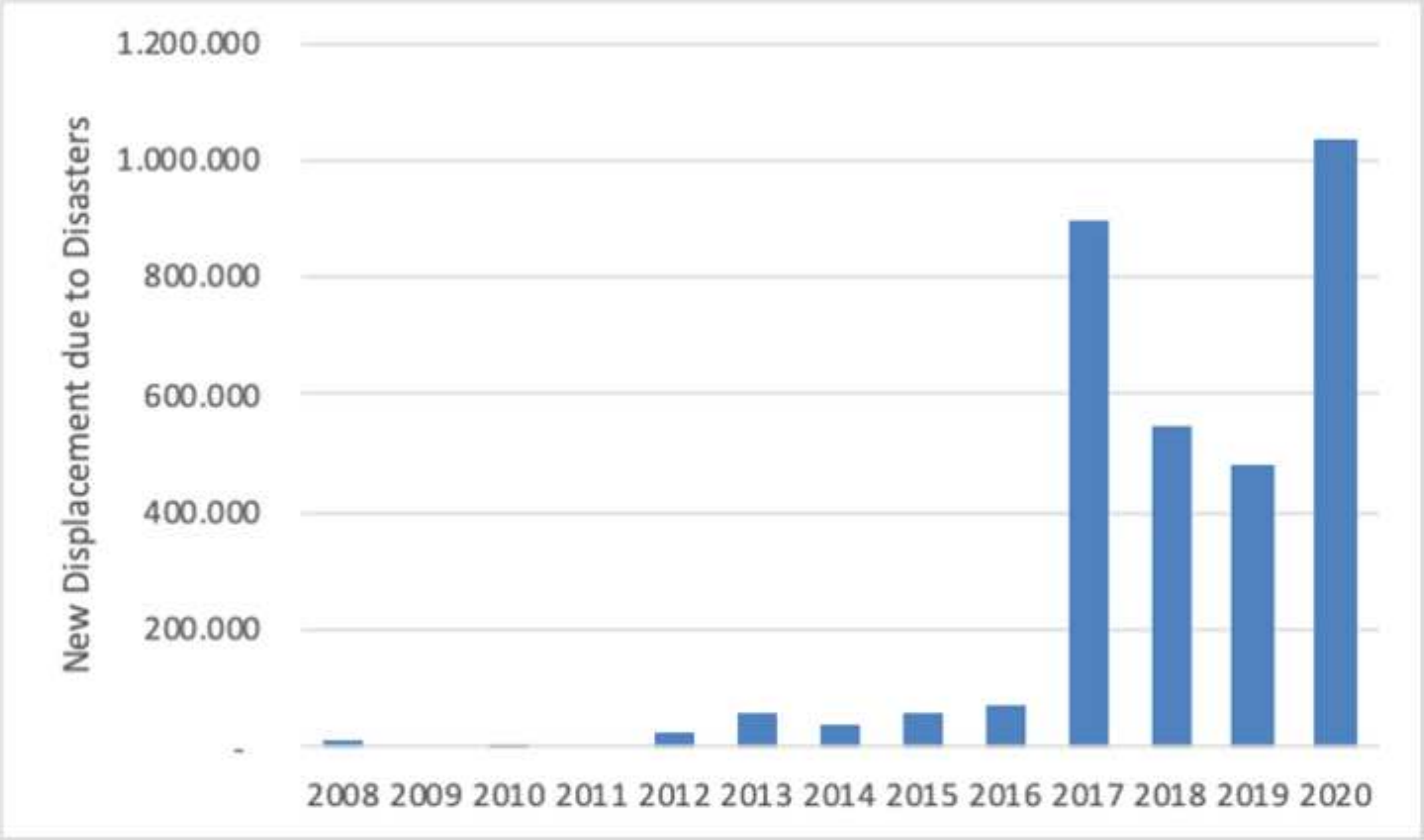

Figure 2

Click here to access/download;Figure;Fig2.tiff

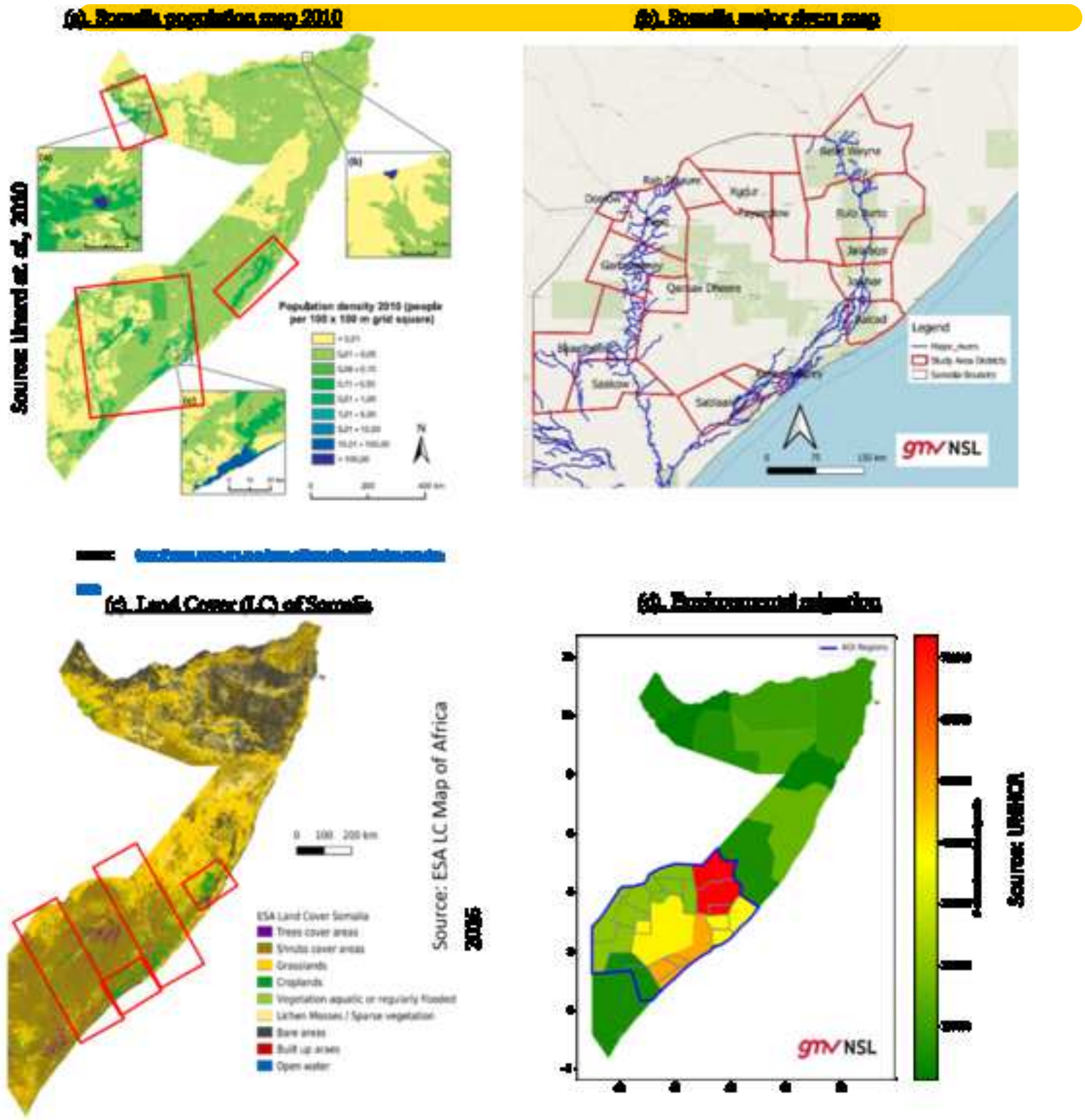

Figure 3

Click here to access/download;Figure;Fig3.tiff

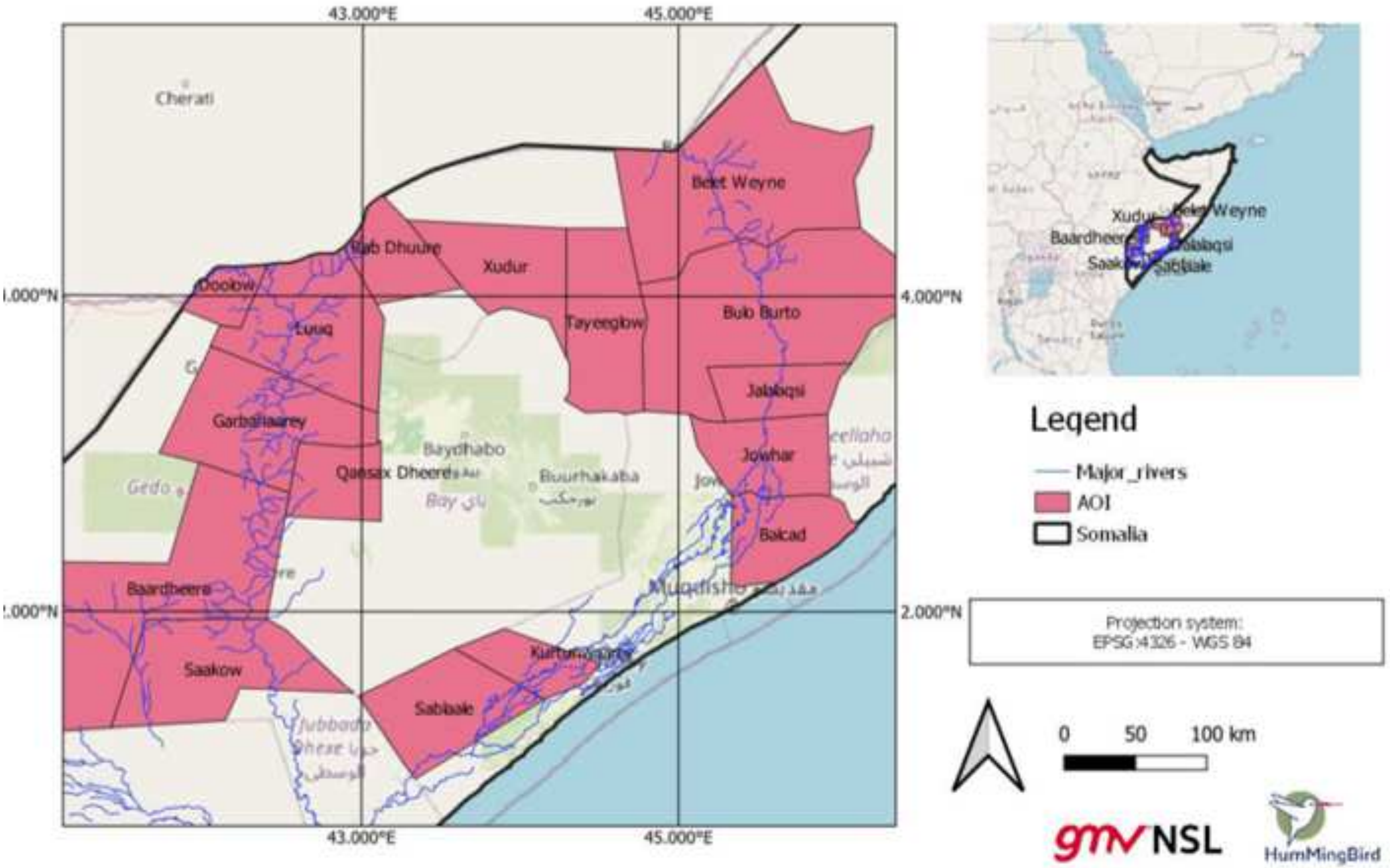

Figure 4

Click here to access/download;Figure;Fig4.tiff

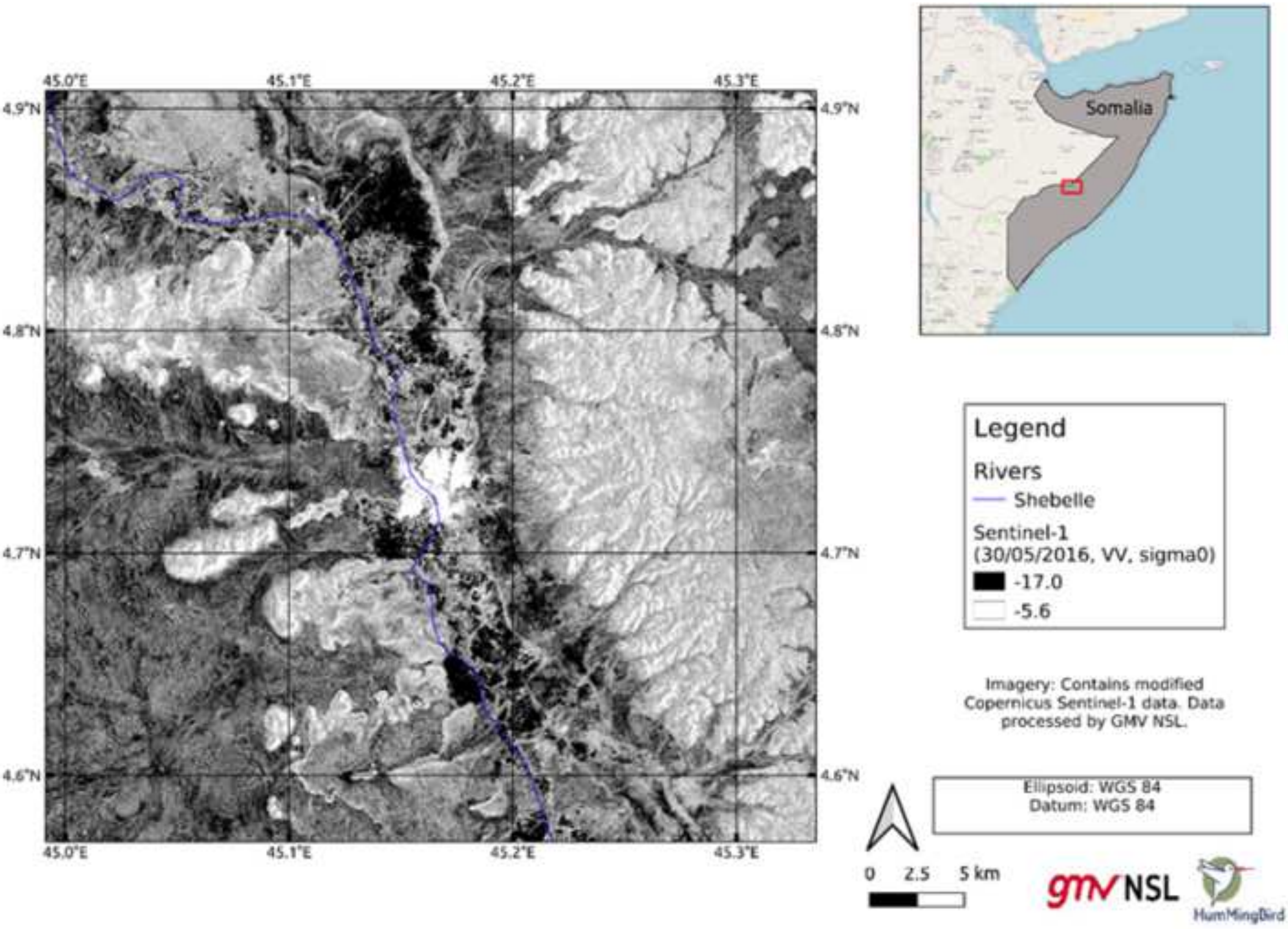

Figure 5

Click here to access/download;Figure;Fig5.tiff

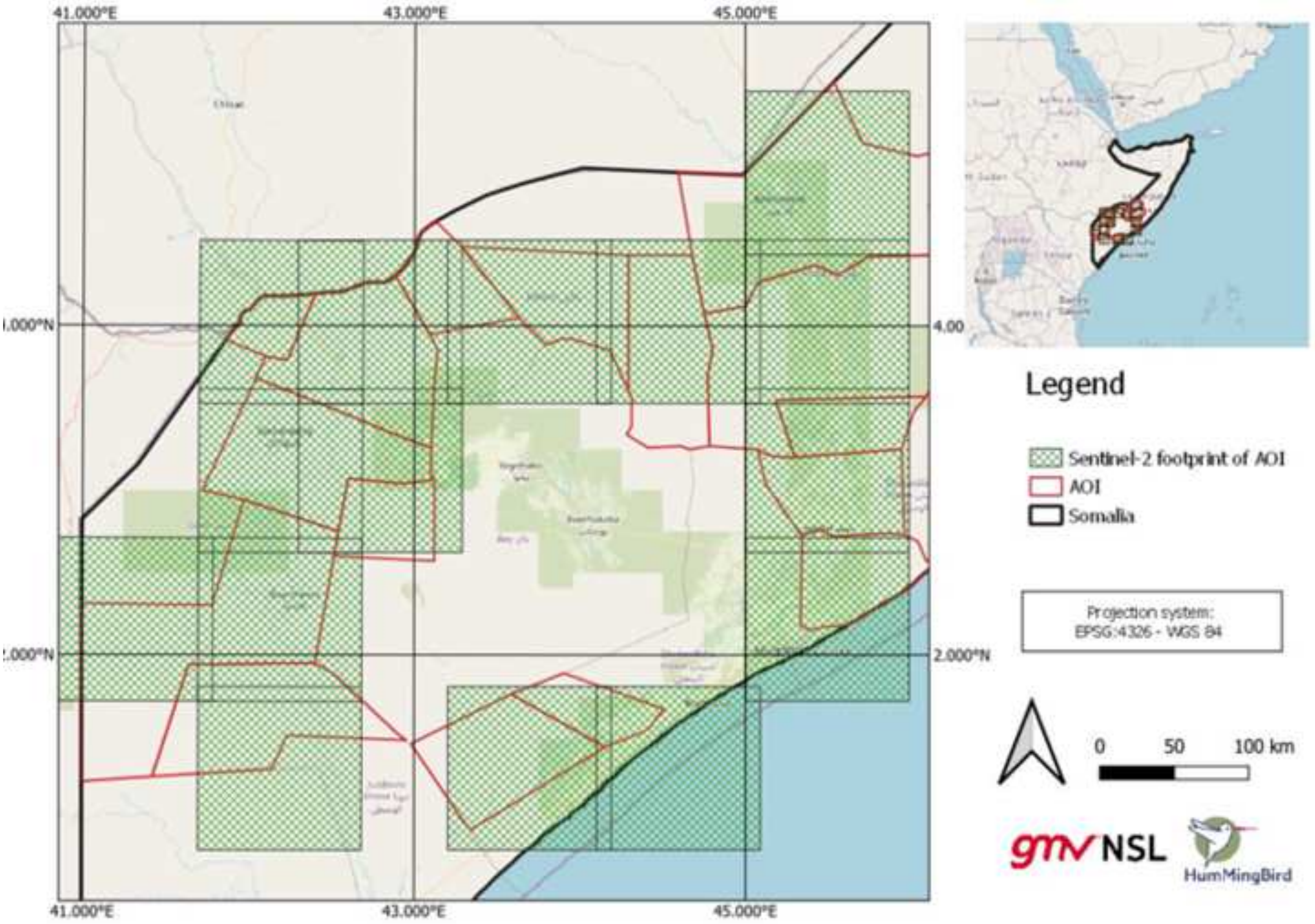

Figure 6

Click here to access/download;Figure;Fig6.tiff

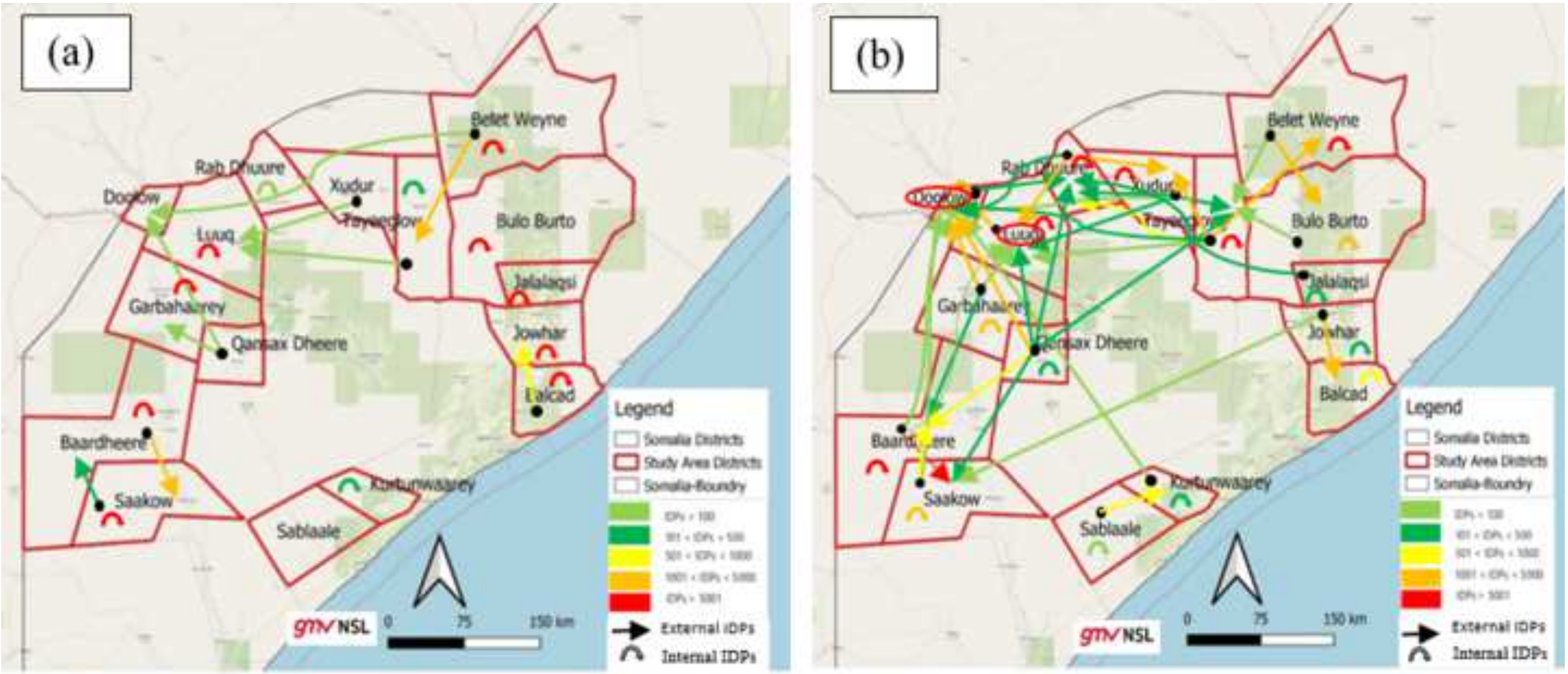

Figure 7

Click here to access/download;Figure;Fig7.tiff

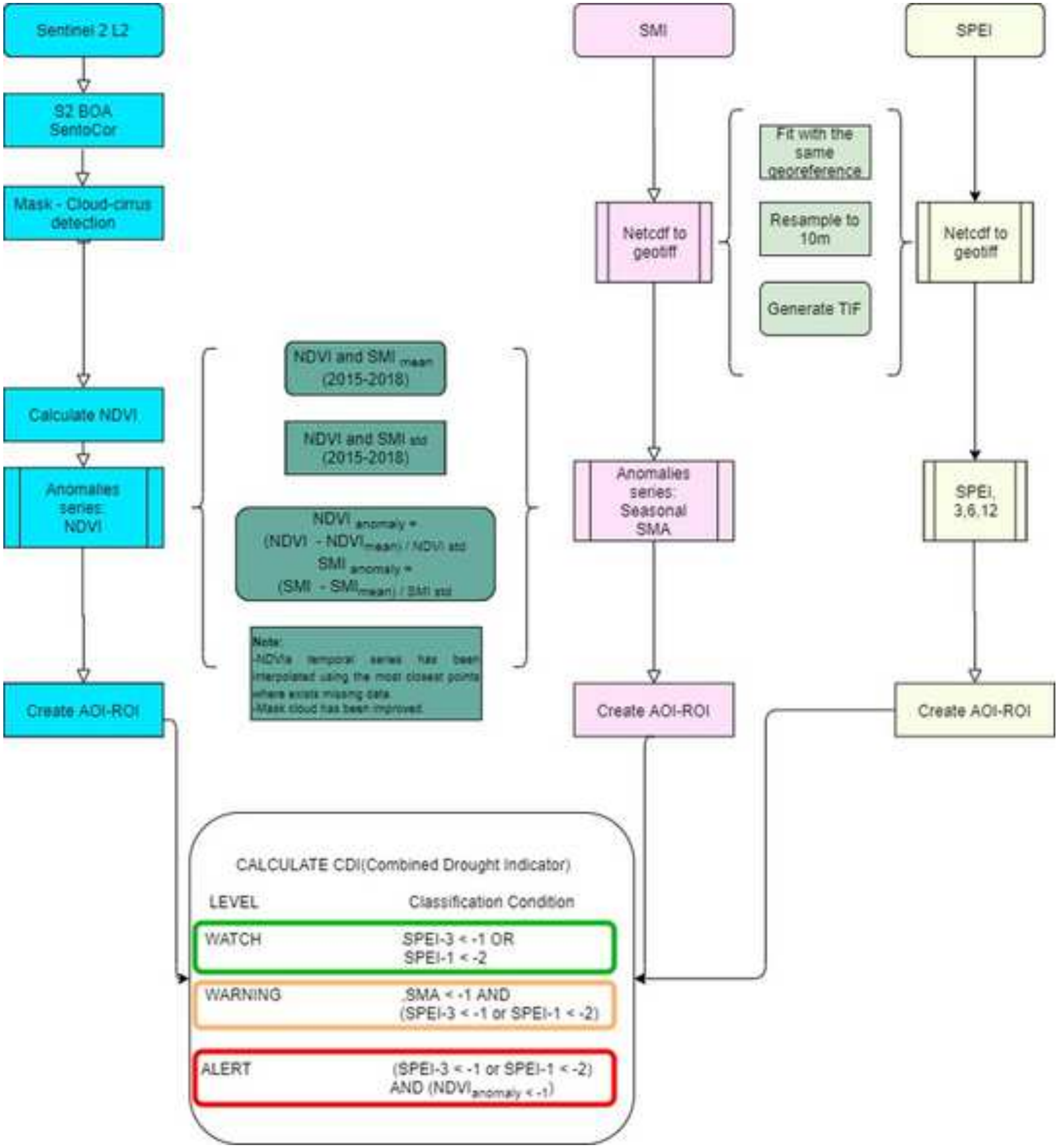

Figure 8

Click here to access/download;Figure;Fig8.tiff

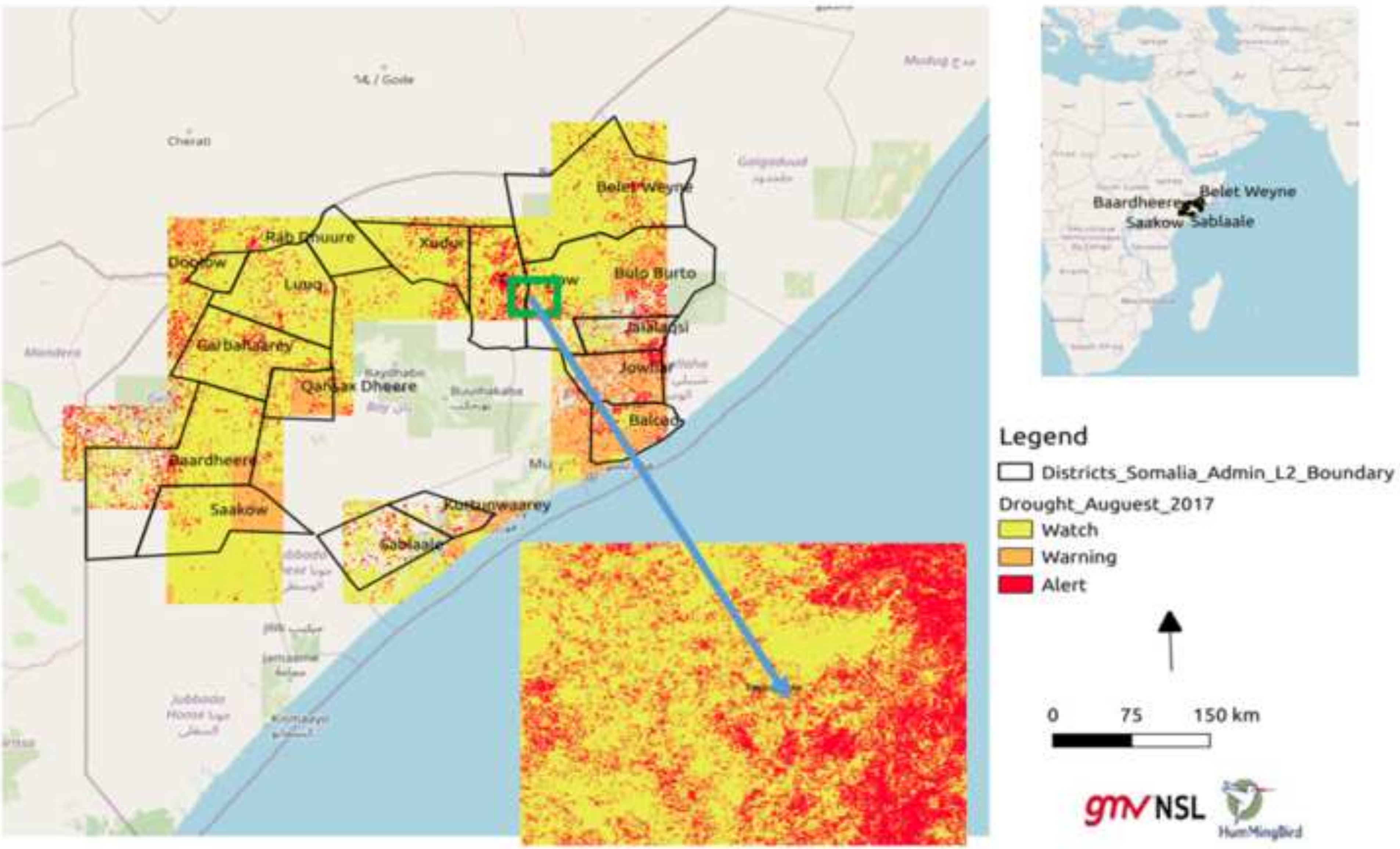

Figure 9

Click here to access/download;Figure;Fig9.tiff

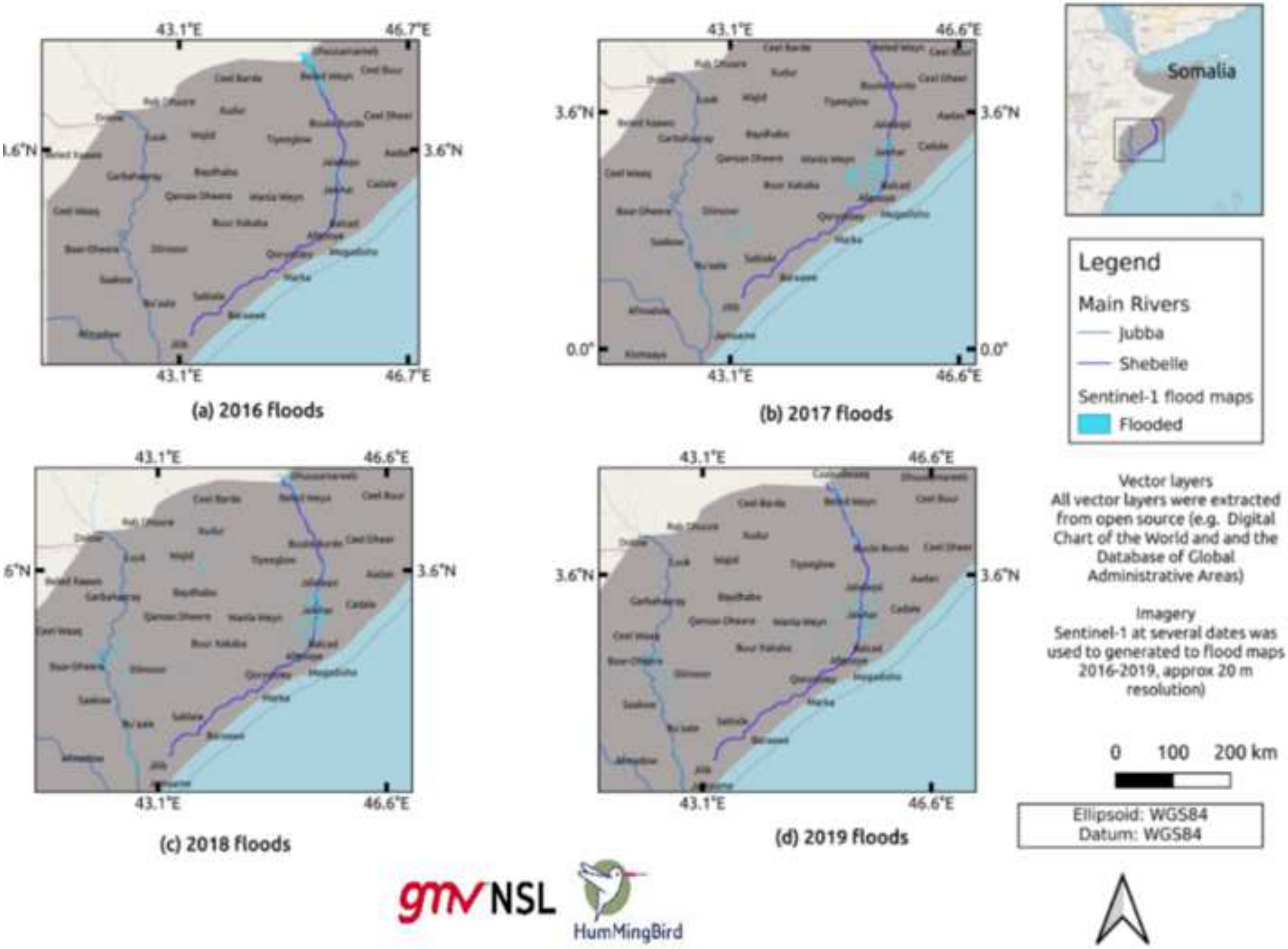

Figure 10

Click here to access/download;Figure;Fig10.tiff

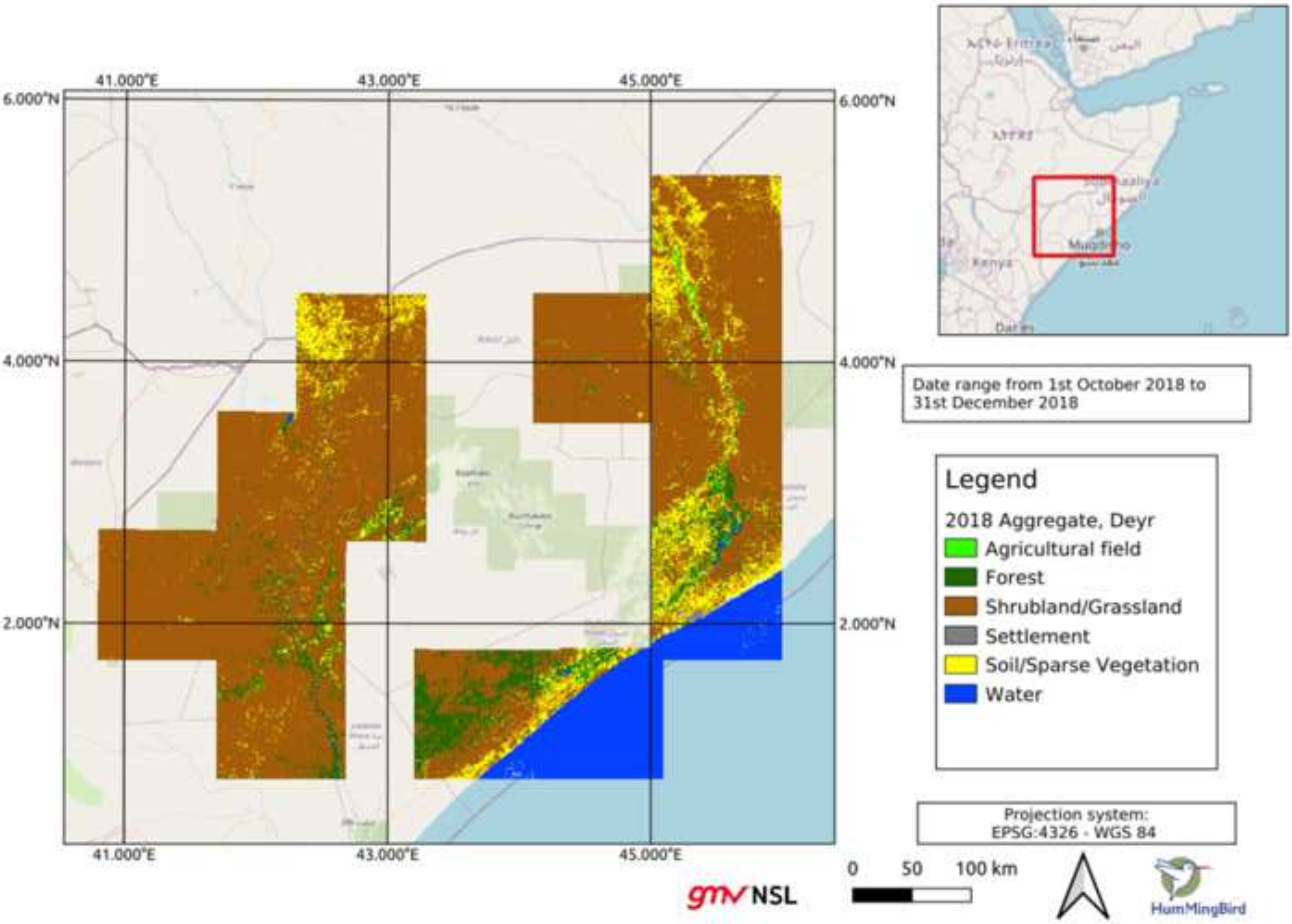

Figure 11

Click here to access/download;Figure;Fig11.tiff

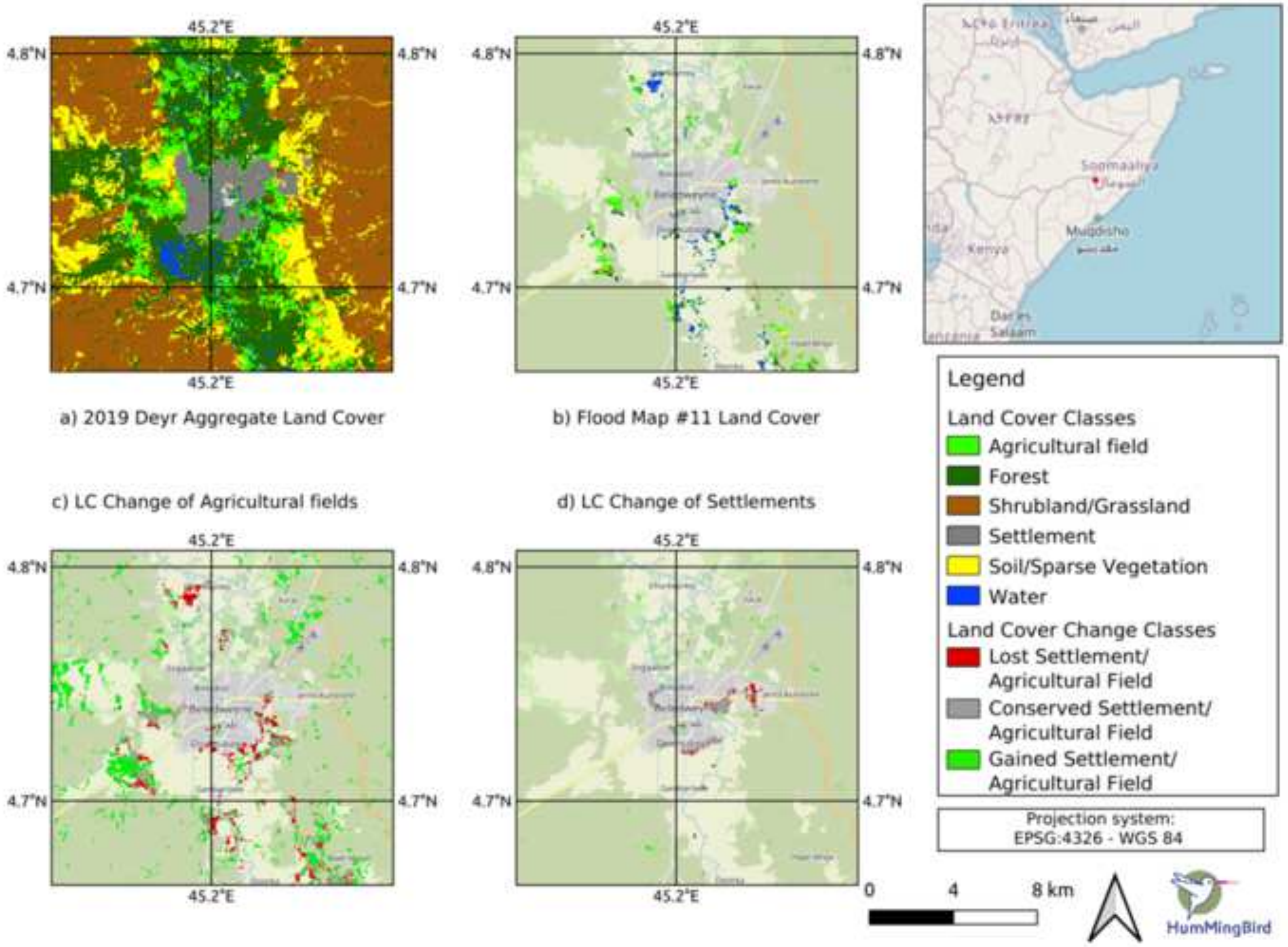

Figure 12

Click here to access/download;Figure;Fig12.tiff

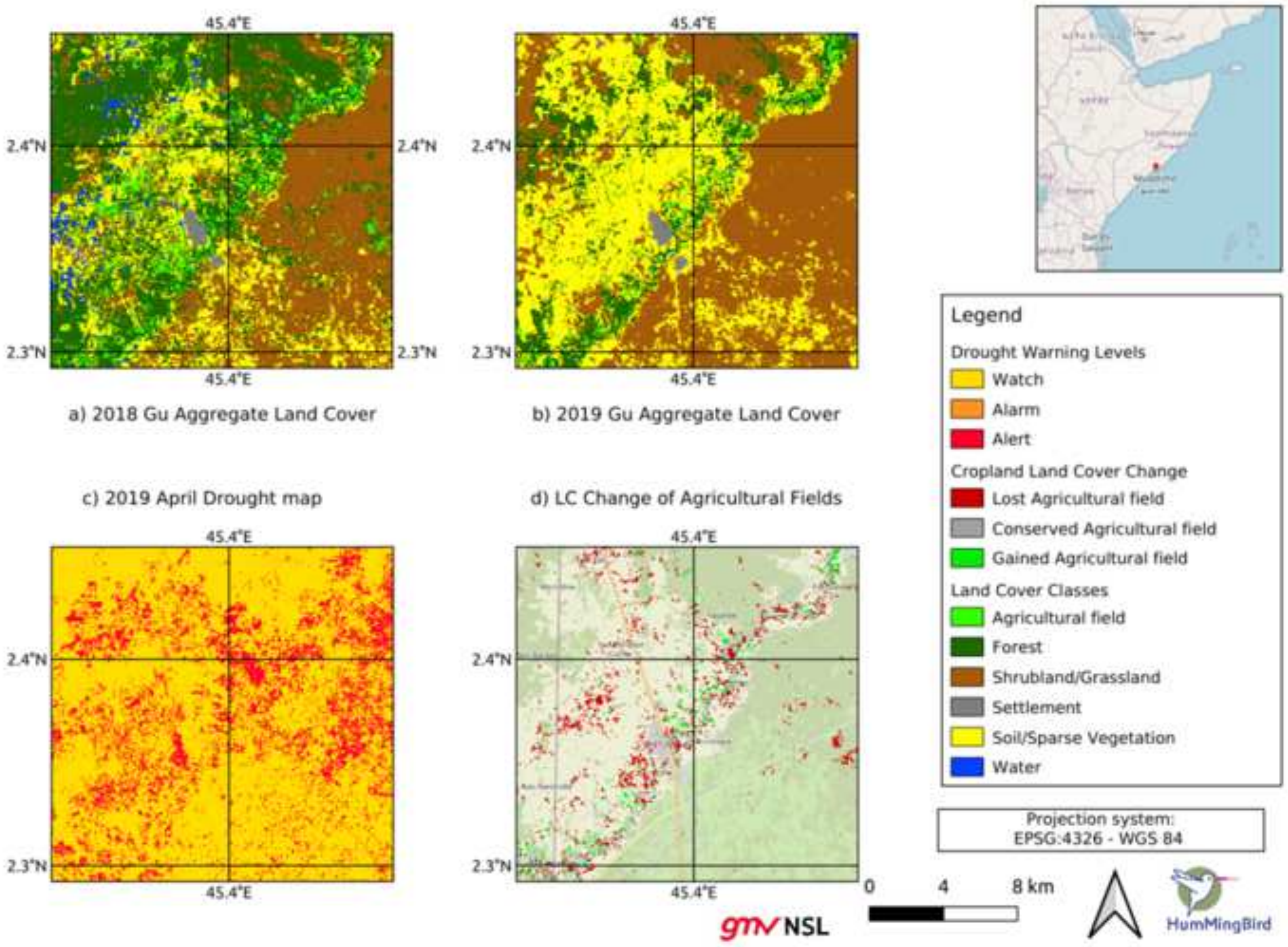

Figure 13

Click here to access/download;Figure;Fig13.tiff

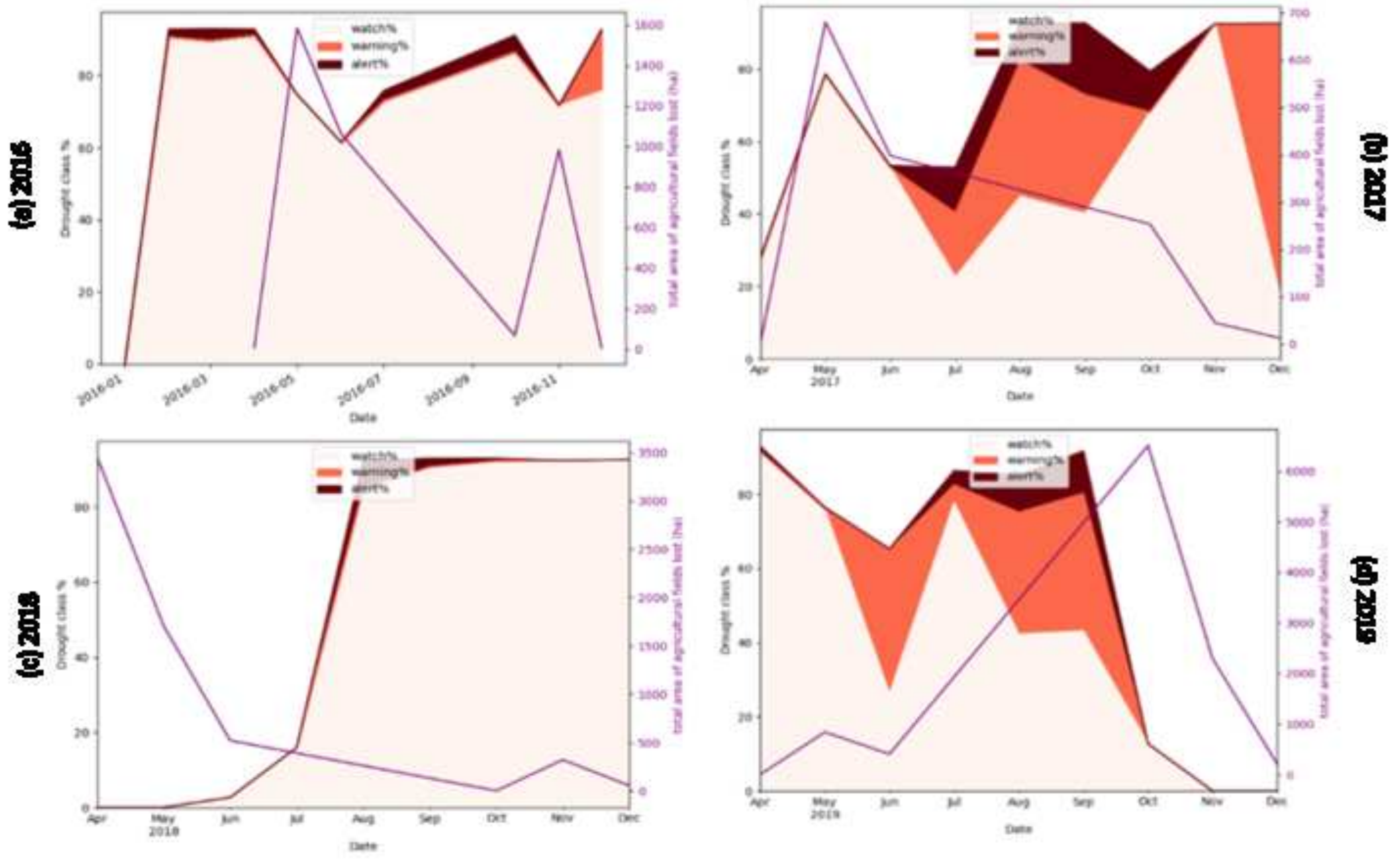

Figure 14

Click here to access/download;Figure;Fig14.tiff

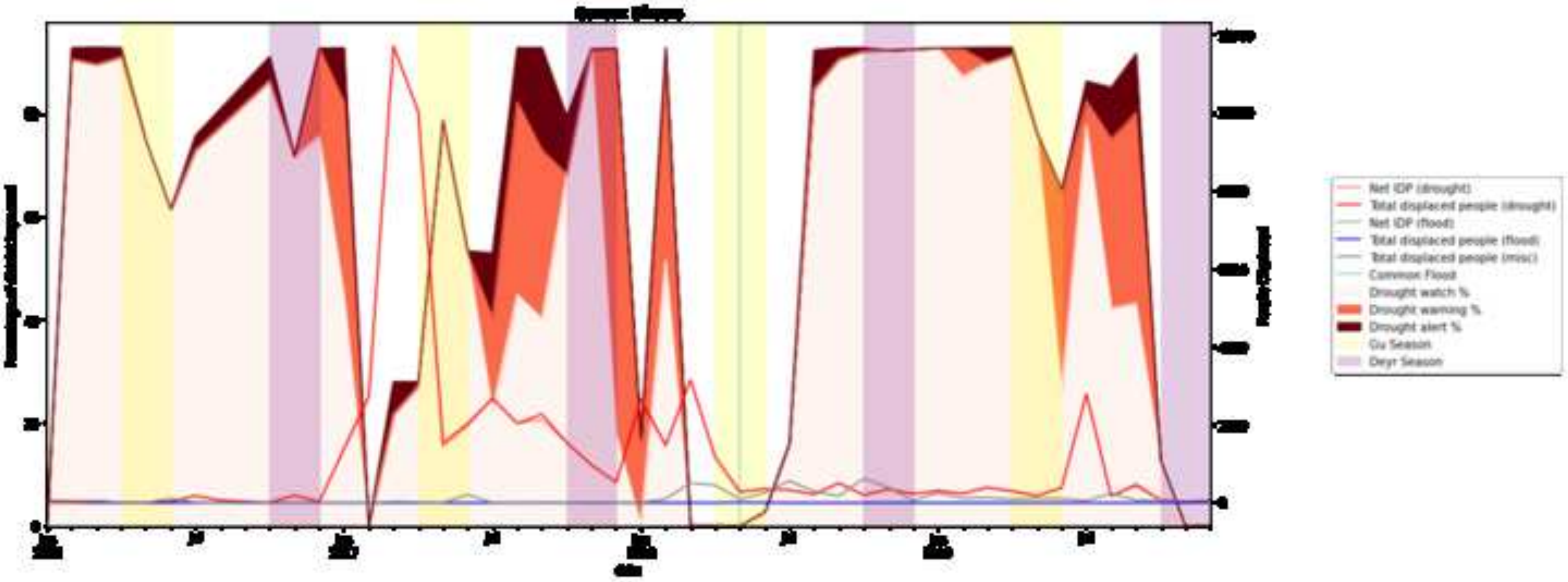

Figure 15

Click here to access/download;Figure;Fig15.tiff

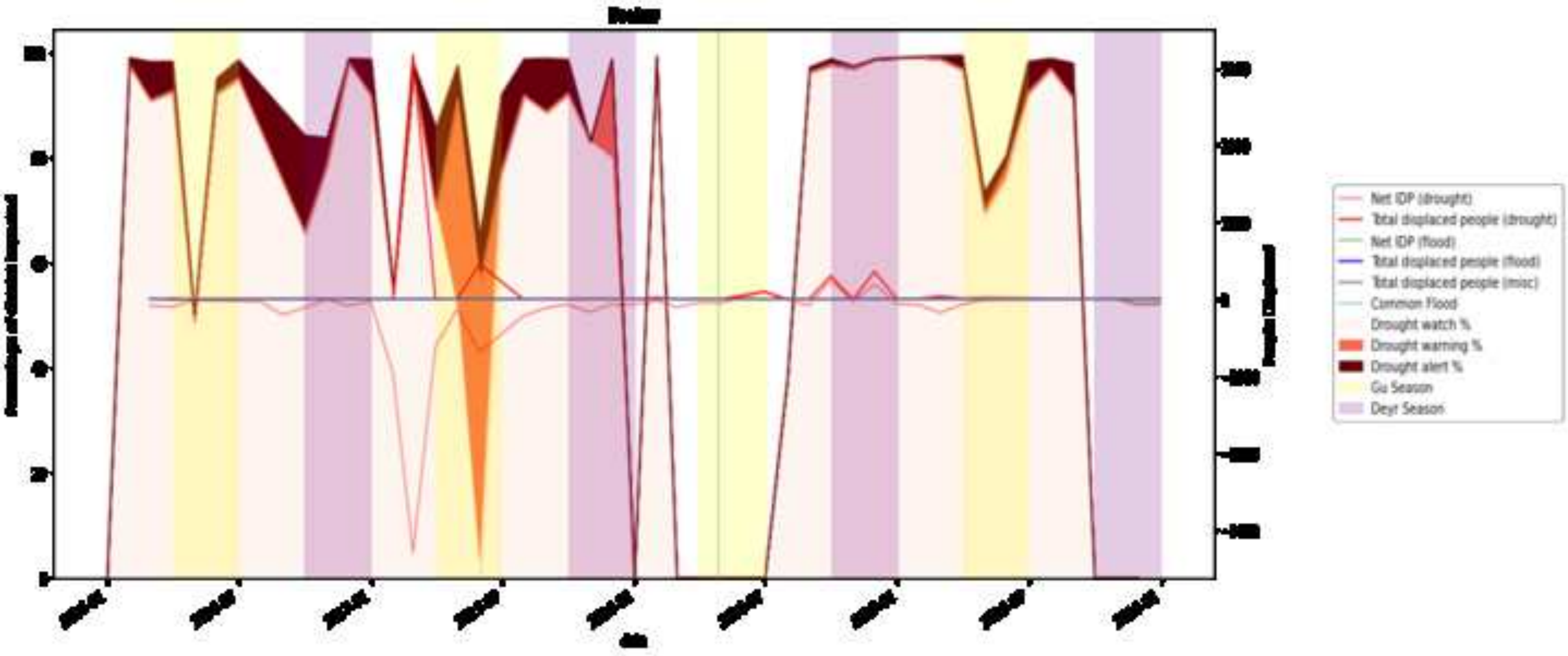

Figure 16

Click here to access/download;Figure;Fig16.tiff

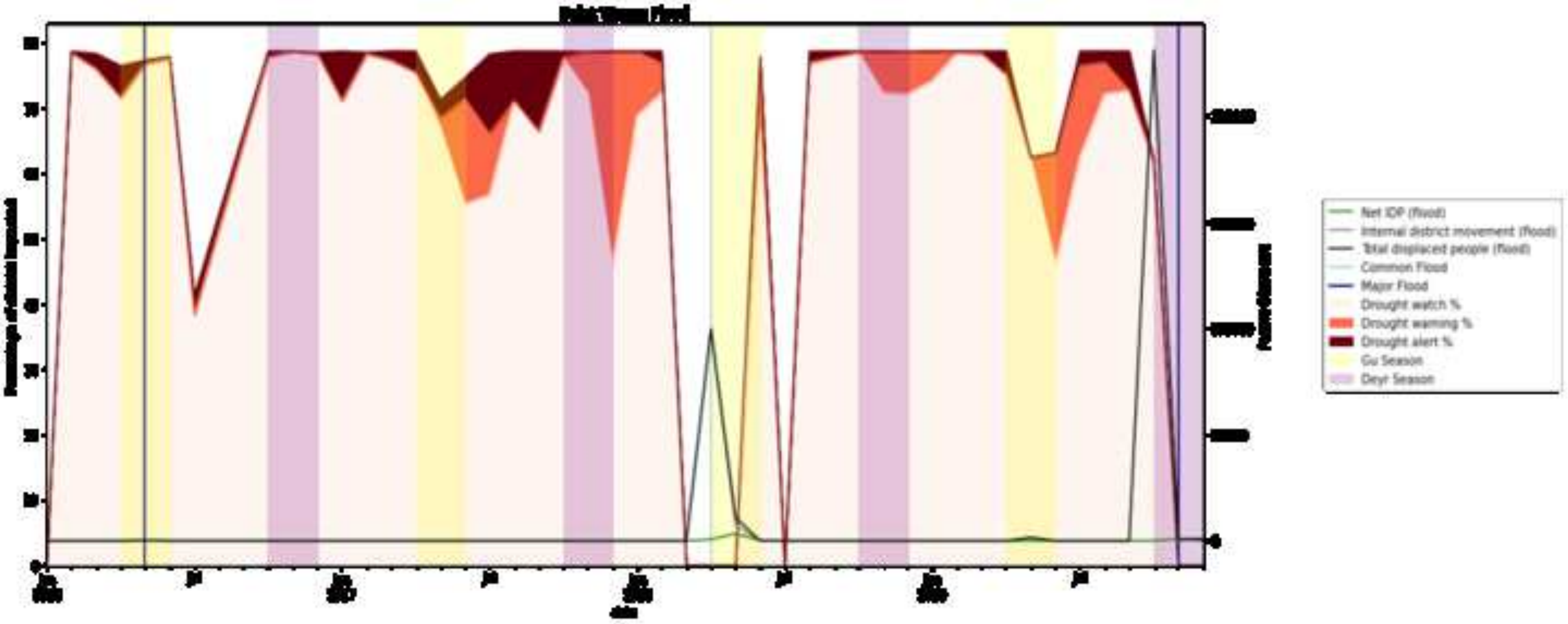

Supplement: Attachment — Submitted filename: PONE-D-23-15881_reveiwed.pdf [file pone.0304202.s001.pdf]
