# Supplementary material for: Deciphering climate-induced displacement in Somalia: A remote sensing perspective
Source: PLoS One. 2024 Aug 9;19(8):e0304202. doi: 10.1371/journal.pone.0304202 (PMC11315326; doi:10.1371/journal.pone.0304202)
Supplement: Attachment — Submitted filename: Rebuttal to the Reviewers.docx [file pone.0304202.s002.docx]

**Deciphering Climate-induced Displacement in Somalia: A Remote Sensing Perspective**

**Response to the Reviewers**

*We would like to thank reviewers for their constructive comments. Please see our response elaborated below for each comment.*

When submitting your revision, we need you to address these additional requirements.

1. Please ensure that your manuscript meets PLOS ONE's style requirements, including those for file naming. The PLOS ONE style templates can be found at 
[https://journals.plos.org/plosone/s/file?id=wjVg/PLOSOne_formatting_sample_main_body.pdf](https://urldefense.com/v3/__https:/journals.plos.org/plosone/s/file?id=wjVg*PLOSOne_formatting_sample_main_body.pdf__;Lw!!MvyJQugb!ACQJu7JFIqNJLGcwL9gQHBj6OJQjv085IEzVZp2PQOY2_6Hjl1N8zteFmq9L_oBPp62tEKb8JGL2FwPx4A$) and 
[https://journals.plos.org/plosone/s/file?id=ba62/PLOSOne_formatting_sample_title_authors_affiliations.pdf](https://urldefense.com/v3/__https:/journals.plos.org/plosone/s/file?id=ba62*PLOSOne_formatting_sample_title_authors_affiliations.pdf__;Lw!!MvyJQugb!ACQJu7JFIqNJLGcwL9gQHBj6OJQjv085IEzVZp2PQOY2_6Hjl1N8zteFmq9L_oBPp62tEKb8JGLoXZ1bjQ$)

*The revised manuscript was controlled for PLOS ONE’s style requirements and updated accordingly.*

2. Thank you for stating the following in the Acknowledgments Section of your manuscript: 
   "This research is supported by the European Commission through the Horizon2020 European project: “HumMingBird – Enhanced migration measures from a multidimensional perspective” (GA: 870661)"

We note that you have provided funding information that is not currently declared in your Funding Statement. However, funding information should not appear in the Acknowledgments section or other areas of your manuscript. We will only publish funding information present in the Funding Statement section of the online submission form. 
Please remove any funding-related text from the manuscript and let us know how you would like to update your Funding Statement. Currently, your Funding Statement reads as follows: 
   "MR, BT, KR

This research is supported by the European Commission through the Horizon2020 European project: “HumMingBird – Enhanced migration measures from a multidimensional perspective” (GA: 870661).

[https://research-and-innovation.ec.europa.eu/funding/funding-opportunities/funding-programmes-and-open-calls/horizon-europe_en](https://urldefense.com/v3/__https:/research-and-innovation.ec.europa.eu/funding/funding-opportunities/funding-programmes-and-open-calls/horizon-europe_en__;!!MvyJQugb!ACQJu7JFIqNJLGcwL9gQHBj6OJQjv085IEzVZp2PQOY2_6Hjl1N8zteFmq9L_oBPp62tEKb8JGIiXqDV4Q$)

The funders had no role in study design, data collection and analysis, decision to publish, or preparation of the manuscript."

Please include your amended statements within your cover letter; we will change the online submission form on your behalf.

*The amended statements are included in the new cover letter.*

3. In your Data Availability statement, you have not specified where the minimal data set underlying the results described in your manuscript can be found. PLOS defines a study's minimal data set as the underlying data used to reach the conclusions drawn in the manuscript and any additional data required to replicate the reported study findings in their entirety. All PLOS journals require that the minimal data set be made fully available. For more information about our data policy, please see [http://journals.plos.org/plosone/s/data-availability](https://urldefense.com/v3/__http:/journals.plos.org/plosone/s/data-availability__;!!MvyJQugb!ACQJu7JFIqNJLGcwL9gQHBj6OJQjv085IEzVZp2PQOY2_6Hjl1N8zteFmq9L_oBPp62tEKb8JGKbEARckg$).

"Upon re-submitting your revised manuscript, please upload your study’s minimal underlying data set as either Supporting Information files or to a stable, public repository and include the relevant URLs, DOIs, or accession numbers within your revised cover letter. For a list of acceptable repositories, please see [http://journals.plos.org/plosone/s/data-availability#loc-recommended-repositories](https://urldefense.com/v3/__http:/journals.plos.org/plosone/s/data-availability*loc-recommended-repositories__;Iw!!MvyJQugb!ACQJu7JFIqNJLGcwL9gQHBj6OJQjv085IEzVZp2PQOY2_6Hjl1N8zteFmq9L_oBPp62tEKb8JGJdjAlYfg$). Any potentially identifying patient information must be fully anonymized.

Important: If there are ethical or legal restrictions to sharing your data publicly, please explain these restrictions in detail. Please see our guidelines for more information on what we consider unacceptable restrictions to publicly sharing data: [http://journals.plos.org/plosone/s/data-availability#loc-unacceptable-data-access-restrictions](https://urldefense.com/v3/__http:/journals.plos.org/plosone/s/data-availability*loc-unacceptable-data-access-restrictions__;Iw!!MvyJQugb!ACQJu7JFIqNJLGcwL9gQHBj6OJQjv085IEzVZp2PQOY2_6Hjl1N8zteFmq9L_oBPp62tEKb8JGJRV7fF1A$). Note that it is not acceptable for the authors to be the sole named individuals responsible for ensuring data access.

We will update your Data Availability statement to reflect the information you provide in your cover letter.

*The data availability) for this specific paper depends on the HumMingBird project (which is the main funding channel for the proposed work) data availability planning. There is a limitation of maximum 52 MB of data can be uploaded into CESSDA platform. However, because of the minimum memory size of each data exceeds 700 MB, the initial products of processed raw data cannot be publicly available. Nevertheless, the aggregated and anonymised data used for this paper is being prepared to be publicly available at Consortium of European Social Science Data Archives (CESSDA) data catalogue and Harvard Dataverse repository as of the end of the HumMingBird project (June 2024).*

4. Please amend the manuscript submission data (via Edit Submission) to include author Robert King.

*The authorship list amended for Robert King. We also added another co-author (Eloy Zafra Santos), as agreed by all existing co-authors.*

5. Please amend your authorship list in your manuscript file to include author Richard King.

*The authorship list amended for Robert King. We also added another co-author (Eloy Zafra Santos), as agreed by all existing co-authors.*

6. We note that Figures 2-6 and 8-12 in your submission contain [map/satellite] images which may be copyrighted. All PLOS content is published under the Creative Commons Attribution License (CC BY 4.0), which means that the manuscript, images, and Supporting Information files will be freely available online, and any third party is permitted to access, download, copy, distribute, and use these materials in any way, even commercially, with proper attribution. For these reasons, we cannot publish previously copyrighted maps or satellite images created using proprietary data, such as Google software (Google Maps, Street View, and Earth). For more information, see our copyright guidelines: [http://journals.plos.org/plosone/s/licenses-and-copyright](https://urldefense.com/v3/__http:/journals.plos.org/plosone/s/licenses-and-copyright__;!!MvyJQugb!ACQJu7JFIqNJLGcwL9gQHBj6OJQjv085IEzVZp2PQOY2_6Hjl1N8zteFmq9L_oBPp62tEKb8JGK0NMA2WA$).
 
We require you to either (a) present written permission from the copyright holder to publish these figures specifically under the CC BY 4.0 license, or (b) remove the figures from your submission:
 
a. You may seek permission from the original copyright holder of Figures 2-6 and 8-12 to publish the content specifically under the CC BY 4.0 license.  
 
We recommend that you contact the original copyright holder with the Content Permission Form ([http://journals.plos.org/plosone/s/file?id=7c09/content-permission-form.pdf](https://urldefense.com/v3/__http:/journals.plos.org/plosone/s/file?id=7c09*content-permission-form.pdf__;Lw!!MvyJQugb!ACQJu7JFIqNJLGcwL9gQHBj6OJQjv085IEzVZp2PQOY2_6Hjl1N8zteFmq9L_oBPp62tEKb8JGIEYG_EFQ$)) and the following text:
“I request permission for the open-access journal PLOS ONE to publish XXX under the Creative Commons Attribution License (CCAL) CC BY 4.0 ([http://creativecommons.org/licenses/by/4.0/](https://urldefense.com/v3/__http:/creativecommons.org/licenses/by/4.0/__;!!MvyJQugb!ACQJu7JFIqNJLGcwL9gQHBj6OJQjv085IEzVZp2PQOY2_6Hjl1N8zteFmq9L_oBPp62tEKb8JGJbqIkp_g$)). Please be aware that this license allows unrestricted use and distribution, even commercially, by third parties. Please reply and provide explicit written permission to publish XXX under a CC BY license and complete the attached form.”
 
Please upload the completed Content Permission Form or other proof of granted permissions as an "Other" file with your submission.
 
In the figure caption of the copyrighted figure, please include the following text: “Reprinted from [ref] under a CC BY license, with permission from [name of publisher], original copyright [original copyright year].”
 
b. If you are unable to obtain permission from the original copyright holder to publish these figures under the CC BY 4.0 license or if the copyright holder’s requirements are incompatible with the CC BY 4.0 license, please either i) remove the figure or ii) supply a replacement figure that complies with the CC BY 4.0 license. Please check copyright information on all replacement figures and update the figure caption with source information. If applicable, please specify in the figure caption text when a figure is similar but not identical to the original image and is therefore for illustrative purposes only.
The following resources for replacing copyrighted map figures may be helpful:
 
USGS National Map Viewer (public domain): [http://viewer.nationalmap.gov/viewer/](https://urldefense.com/v3/__http:/viewer.nationalmap.gov/viewer/__;!!MvyJQugb!ACQJu7JFIqNJLGcwL9gQHBj6OJQjv085IEzVZp2PQOY2_6Hjl1N8zteFmq9L_oBPp62tEKb8JGLlTk-0rg$)
The Gateway to Astronaut Photography of Earth (public domain): [http://eol.jsc.nasa.gov/sseop/clickmap/](https://urldefense.com/v3/__http:/eol.jsc.nasa.gov/sseop/clickmap/__;!!MvyJQugb!ACQJu7JFIqNJLGcwL9gQHBj6OJQjv085IEzVZp2PQOY2_6Hjl1N8zteFmq9L_oBPp62tEKb8JGJEdoSkbw$)
Maps at the CIA (public domain): [https://www.cia.gov/library/publications/the-world-factbook/index.html](https://urldefense.com/v3/__https:/www.cia.gov/library/publications/the-world-factbook/index.html__;!!MvyJQugb!ACQJu7JFIqNJLGcwL9gQHBj6OJQjv085IEzVZp2PQOY2_6Hjl1N8zteFmq9L_oBPp62tEKb8JGIrosDPSA$) and [https://www.cia.gov/library/publications/cia-maps-publications/index.html](https://urldefense.com/v3/__https:/www.cia.gov/library/publications/cia-maps-publications/index.html__;!!MvyJQugb!ACQJu7JFIqNJLGcwL9gQHBj6OJQjv085IEzVZp2PQOY2_6Hjl1N8zteFmq9L_oBPp62tEKb8JGILkWWB6w$)
NASA Earth Observatory (public domain): [http://earthobservatory.nasa.gov/](https://urldefense.com/v3/__http:/earthobservatory.nasa.gov/__;!!MvyJQugb!ACQJu7JFIqNJLGcwL9gQHBj6OJQjv085IEzVZp2PQOY2_6Hjl1N8zteFmq9L_oBPp62tEKb8JGKDUY8KPw$)
Landsat: [http://landsat.visibleearth.nasa.gov/](https://urldefense.com/v3/__http:/landsat.visibleearth.nasa.gov/__;!!MvyJQugb!ACQJu7JFIqNJLGcwL9gQHBj6OJQjv085IEzVZp2PQOY2_6Hjl1N8zteFmq9L_oBPp62tEKb8JGI91-EI-g$)
USGS EROS (Earth Resources Observatory and Science (EROS) Center) (public domain): [http://eros.usgs.gov/#](https://urldefense.com/v3/__http:/eros.usgs.gov/*__;Iw!!MvyJQugb!ACQJu7JFIqNJLGcwL9gQHBj6OJQjv085IEzVZp2PQOY2_6Hjl1N8zteFmq9L_oBPp62tEKb8JGI1lXKACg$)
Natural Earth (public domain): [http://www.naturalearthdata.com/](https://urldefense.com/v3/__http:/www.naturalearthdata.com/__;!!MvyJQugb!ACQJu7JFIqNJLGcwL9gQHBj6OJQjv085IEzVZp2PQOY2_6Hjl1N8zteFmq9L_oBPp62tEKb8JGJANa2t8g$)

*All figures are recreated and all logos that might create a copyright issues were removed from the figures.*

7. Please include a separate caption for each figure in your manuscript.

*Separate captions and relevant legends are included within the text for all figures.*

[Note: HTML markup is below. Please do not edit.]

Reviewers' comments:

Reviewer's Responses to Questions

**Comments to the Author**

1. Is the manuscript technically sound, and do the data support the conclusions?

The manuscript must describe a technically sound piece of scientific research with data that supports the conclusions. Experiments must have been conducted rigorously, with appropriate controls, replication, and sample sizes. The conclusions must be drawn appropriately based on the data presented.

Reviewer #1: Partly

Reviewer #2: Yes

2. Has the statistical analysis been performed appropriately and rigorously?

Reviewer #1: No

Reviewer #2: No

3. Have the authors made all data underlying the findings in their manuscript fully available?

The [PLOS Data policy](https://urldefense.com/v3/__http:/www.plosone.org/static/policies.action*sharing__;Iw!!MvyJQugb!ACQJu7JFIqNJLGcwL9gQHBj6OJQjv085IEzVZp2PQOY2_6Hjl1N8zteFmq9L_oBPp62tEKb8JGKqM6dbvg$) requires authors to make all data underlying the findings described in their manuscript fully available without restriction, with rare exception (please refer to the Data Availability Statement in the manuscript PDF file). The data should be provided as part of the manuscript or its supporting information, or deposited to a public repository. For example, in addition to summary statistics, the data points behind means, medians and variance measures should be available. If there are restrictions on publicly sharing data—e.g. participant privacy or use of data from a third party—those must be specified.

Reviewer #1: No

Reviewer #2: No

4. Is the manuscript presented in an intelligible fashion and written in standard English?

PLOS ONE does not copyedit accepted manuscripts, so the language in submitted articles must be clear, correct, and unambiguous. Any typographical or grammatical errors should be corrected at revision, so please note any specific errors here.

Reviewer #1: No

Reviewer #2: Yes

5. Review Comments to the Author

Please use the space provided to explain your answers to the questions above. You may also include additional comments for the author, including concerns about dual publication, research ethics, or publication ethics. (Please upload your review as an attachment if it exceeds 20,000 characters)

Reviewer #1: The work has a good theme but needs changes:

* Needs a grammatical review, giving special attention to words, such as line 5 (acknowledgement), line 14 (emphasise), line 25 (neighbouring), and so on.

*Thanks for pointing out the need for language improvement. The revised text does not only address content related comments, but it was also edited to ensure high quality language use in writing. Accordingly, the revised version has gone through a thorough language editing (for British English) to improve the language and the narrative. All abbreviations were used only after the full names/text were introduced first. Difficult to understand sentences are rewritten. Besides, the full text was proofread by a native speaker to avoid typos and grammatical mistakes.*

* The text has several statements, which do not contemplate the results of the work, without a theoretical basis (citations). Bringing more citations in the text is of paramount importance.

*The text is revised and further and more recent scientific citations are added.*

* The research addresses climate change, but its study period is from 2016 to 2019, which under no circumstances represents climate change. Other satellites, in addition to the sentinels, could be used for at least another 25 years, with migration data available from 2015 onwards. The union of these data would contribute to the SMI and SPEI data.

*Thank you for your insightful remark on the study period and the potential use of other satellites to extend our temporal scope.*

*While the study period of 2016 to 2019 may seem limited in the context of climate change, our focus was on utilising the Sentinel series for its unique attributes and high-resolution data. Our methodology involved the development of unique indicators from scratch, rather than employing pre-existing products or indicators. This choice, although presenting certain advantages in the precision and relevance of our findings, introduced significant complexities.*

*Merging diverse data sources from satellites other than the Sentinels becomes especially challenging when creating custom indicators, as opposed to using off-the-shelf products. Different satellites often possess varying spatial and spectral resolutions, calibration systems, and other sensor-specific traits. Integrating these diverse datasets for a seamless and scientifically robust output required meticulous calibration, correction, and harmonisation processes, which, given our specific approach, could have introduced inconsistencies and uncertainties.*

*Furthermore, the processing of such vast and diverse datasets is not only technically challenging but also immensely time-consuming. Our indicators, being unique, required tailored pre-processing and analysis steps, each of which demands substantial computational resources and time. Extending our study period to incorporate another 25 years of data would have multiplied the processing time and could have jeopardised the timely completion of the study.*

*That said, we recognise the value in a more extended temporal analysis to capture the nuances of climate change more comprehensively. We are keen to delve deeper into this in future projects, possibly by collaborating with experts in satellite data harmonisation, or by employing more advanced computational techniques and resources.*

* It remains to elucidate in the introduction how remote sensing can contribute to studying the relationship between the climatic indicator and the displacement of people.

*The introduction is revised and we added a new paragraph (p.1) to demonstrate the significance of remote sensing to investigate the climate indicators and human displacement.*

* The formulas in lines 327, 333, 338, 356, and 447 do not contain references.

*The message “*Error! Reference source not found.*” Appear where anchoring to the referred figures and maps failed to appear when the submitted Word file is converted to pdf by the submission system. The anchors are removed to avoid such error messages. Formulas are also updated and revised accordingly.*

* Lines 472, 519, 539, 554, 562, 568, 587, 591, and 603 also do not contain references.

*The message “*Error! Reference source not found.*” Appear where anchoring to the referred figures and maps failed to appear when the submitted Word file is converted to pdf by the submission system. The anchors are removed to avoid such error messages.*

* In the discussions, you also need some references to confirm the n statements made (ex: line 689). As well as to represent an honest discussion and not just a summary of the work.

*Thank you for pointing out the oversight in our discussion section. We understand the importance of substantiating our statements with credible references to lend more weight and credibility to our assertions. In response to your comment:*

*We thoroughly reviewed the discussion section, especially at the mentioned line (line 689), to identify instances where claims or statements can be strengthened with appropriate references from the existing body of knowledge.*

*We also acknowledge that the discussion section should not merely be a summary of the work but should delve deeper into the implications, comparisons, and relevance of our findings in the broader context of the field. To address this, we integrated new text (p.35) with scientific references from the field on more comparative and critical analyses, drawing parallels and distinctions with established research in the field, and provide insights into the broader significance of our findings.*

* The maps in Figures 2,6, and 8 need geolocalized coordinates.

*Thank you for highlighting the necessity for geolocalised coordinates on the maps in Figures 2, 6, and 8. If by "geolocalised coordinates" the reviewers are referring to the addition of clear extent coordinates, we have indeed addressed this for the corresponding maps:*

*For the Area of Interest (AOI): The extent coordinates using the projection system WGS 84 (EPSG:4326) are 41, 0.3 to 47, 5.4.*

*For Somalia: The extent coordinates using the projection system WGS 84 (EPSG:4326) are 41, -1.7 to 51.4, 12.*

*We hope this addition enhances the clarity and utility of our maps.*

* All figures are without description.

*Thanks for pointing out the oversight regarding the figure descriptions. We acknowledge the importance of detailed figure captions to provide context and clarity to the readers. We revised the manuscript to include comprehensive descriptions for all figures to ensure they effectively convey the intended information.*

Reviewer #2: In this study the optical (S-2) and SAR (S-1) images were used along with some other environmental data such as soil moisture, precipitation-evapotranspiration, and digital elevation model to develop climate indicators which were utilized together with some socio-economic indicators to study the relationship between climate factors and internally displaced persons in a district level in Somalia.

A relatively high amount of technical work has been done, which makes the paper technically acceptable. However, there are still shortcomings such as the lack of quantitative assessment of the accuracy of the classification results. Please find the attached file to see all my comments and suggestions.

*Thanks to the reviewer for acknowledging the extensive technical work invested in the paper. We appreciate their feedback and the attached detailed comments.*

*We’d like to start with the remark about the potential benefits of utilising target decomposition features, particularly with respect to making use of data across all polarisations rather than relying solely on VV polarisation. While the recommendation is indeed valuable, our primary focus for this study revolves around assessing changes in specific land use types, notably human settlements and agricultural fields, as direct consequences of climate-related disasters. This perspective guided our decision to prioritise certain methodologies over others. However, we acknowledge the merit of your suggestion, and it offers an avenue for more in-depth analyses in future studies where the emphasis could be expanded beyond our current scope.*

*Regarding the quantitative assessment of classification results, we have indeed incorporated an accuracy assessment for Land Cover (LC). We recognise the importance of comprehensive accuracy assessments. While we can extend the accuracy analysis to drought and flood classifications, it's worth noting that such an inclusion would be substantial. Given the magnitude and potential depth of this additional analysis, we believe it might be more suitable for a separate paper that can delve deeper into the specific nuances of drought and flood classifications. We genuinely value their suggestion, and we will certainly consider it for future research and publication. Their insights are helping to shape our next steps in this area of study.*

*Moreover, among the reviewer’s comments, they astutely highlighted the potential of full-polarisation SAR data, emphasising the efficacy of polarimetric techniques such as "target decomposition" over raw polarised data (i.e., vv, HH, and HV/VH) in detecting specific objects. While the application of such techniques certainly holds promise for enhancing accuracy, it was beyond the purview of the current study. Nevertheless, we recognise the merit of this suggestion. Future investigations with a similar application will indeed consider incorporating these advanced polarimetric techniques to further refine the results and provide a more comprehensive analysis. We addressed this comment by adding this suggestion as a technical limitation and hence a potential for directions for future research (p.37).*

*Last but not least, in response to their feedback regarding the necessity for rigorous accuracy assessment of classification results, we have now incorporated a comprehensive accuracy assessment in our manuscript. Specifically, we've added Table 5 (p.25), which presents the confusion matrix elucidating the comparisons between our classification results and ground truth data. Furthermore, we have expanded our discussion in a new paragraph dedicated to accuracy considerations, detailing not only the overall accuracy but also other significant metrics such as the kappa coefficient. We trust that these additions provide a more transparent and rigorous evaluation of our classification outcomes, aligning with best practices in the field.*
