# Supplementary material for: Deciphering climate-induced displacement in Somalia: A remote sensing perspective
Source: PLoS One. 2024 Aug 9;19(8):e0304202. doi: 10.1371/journal.pone.0304202 (PMC11315326; doi:10.1371/journal.pone.0304202)
Supplement: Attachment — Submitted filename: Response to the Reviewers_Final.docx [file pone.0304202.s003.docx]

**Deciphering Climate-induced Displacement in Somalia: A Remote Sensing Perspective**

**Response to the Reviewers - Final**

We would like to thank reviewers for their constructive comments. In addition to all prior revision, we addressed all final (mostly minor) remarks of the reviewers.
